# The recent progress in the catalytic conversion of nitroarene into amino arene catalyzed by heterogeneous metal based nano catalyst

**DOI:** 10.3389/fchem.2026.1827073

**Published:** 2026-06-09

**Authors:** Tabassum Manzoor, Mazloom Shah, Sohail Ahmed, Muhammad Imran Jamil, Shahid Iqbal, Sajid Mahmood, Salah Knani, Reem Alreshidi

**Affiliations:** 1 Department of Chemistry, Faculty of Science, Grand Asian University, Sialkot, Pakistan; 2 Department of Chemical and Environmental Engineering, University of Nottingham Ningbo China, Ningbo, China; 3 Low Dimensional Materials Research Center at Khazar University, Baku, Azerbaijan; 4 Center for Scientific Research and Entrepreneurship, Northern Border University, Arar, Saudi Arabia; 5 Department of Physics, College of Science, Northern Border University, Arar, Saudi Arabia

**Keywords:** amino arenes, heterogenous nanocatalysts, hydrogen source, nitroarenes, noble metals, non-noble metals, reduction

## Abstract

An overview of how heterogeneous metal-based nanocatalysts can hydrogenate nitroarenes to amino arenes has drawn a lot of interest because of its industrial significance as an intermediate in the production of plastics, as well as for the pharmaceutical, agrochemical and material sciences industries. Novel nanocatalysts, including noble (Pd, Pt, Au, Ru, Rh and Ag) and non-noble (Cu, Fe, Ni, Co and Mn) metal nanoparticles supported on carbonaceous materials, metal-organic frameworks (MOFs) and metal oxides, have been used in recent years to improve catalytic efficiency, selectivity and sustainability. Bimetallic alloys, single-atom catalysts (SACs) and plasmonic nanomaterials are important advancements that allow for effective nitro reduction using hydrogen (H_2_), hydrazine (N_2_H_2_), sodium borohydride (NaBH_4_) or photocatalysis in mild conditions. Depending on the type of catalyst, mechanistic details show different paths and environmentally friendly techniques including solvent-free reactions, electrochemical reduction, and magnetic recoverable catalysts support sustainable synthesis. Recent developments in heterogeneous metal-based nanocatalysts for nitroarene reduction are thoroughly compiled in this study, which emphasizes the critical transformation from noble to non-noble metal systems that provide more affordable and environmentally friendly alternatives. Despite advancements, issues with scalability, cost containment, and long-term stability still exist. This review also offers a roadmap for further study in this ground-breaking subject by highlighting current advances in nanocatalyst engineering, reaction mechanisms and upcoming tactics.

## 1 Introduction

**SCHEME 1:**
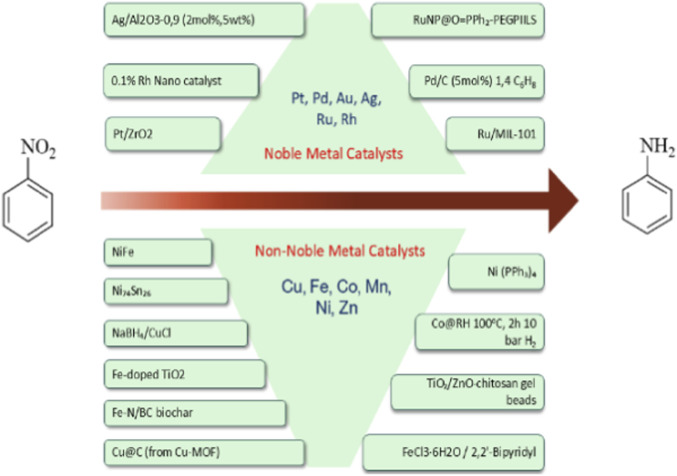
Schematic diagram for conversion of nitrobenzene into amino benzene catalyzed by various heterogeneous nano catalysts.

Since the industrial era, when nitroarenes were first released into the environment through the manufacture of explosives, dyes and other industrial chemicals, these substances have been an hazardous due to their extreme toxicity and environmental persistence. Their environmental presence has been connected to a lot of health concerns such as respiratory disorders and cancer. To minimize their harmful consequences there has been a growing interest in developing methods to remove nitroaromatic contaminants from the environment (Anusuya et al., 2021; Wang et al., 2025; Wang and Zhang, 2014). In synthetic organic chemistry, one of the most important processes is the conversion of nitro compounds to their equivalent anilines. Aromatic amines are used as intermediates in the preparation of significant commercial products, such as dyes, pharmaceuticals, and agricultural chemicals. (Raimundo et al., 2023; Tavakolian et al., 2025). The reduction of aromatic compounds by nitro groups using NaBH_4_ in the presence of metal catalysts has recently received a lot of attention due to the significant catalytic activity of metal particles (Bahadur et al., 2025; Zirak et al., 2025). Aromatic amines serve as vital beginning and intermediate products in the chemical manufacture of numerous industry sectors such as dye, pigment, agrochemical, polymer, and medicine (Genc, 2015; Yao et al., 2025). Currently, the catalytic reduction of nitrobenzene requires innovative, affordable and efficient catalysts. A new copper-based metal-organic framework (MOF) denoted as Cu@C, has been reported by calcining Cu–MOF at 700 °C. MOF confinement (2–5 nm) prevents aggregation; enables tandem catalysis. With (NaBH_4_) as the reducing agent, the catalyst showed outstanding catalytic performance in reducing nitrobenzene, exhibiting a high conversion rate of 100% and a quick reaction time of 8 min (Tang et al., 2023; Zhang S. et al., 2025). Recent advancements in catalysts using inexpensive metals such as Fe, Co, Ni, and Cu have produced remarkable results, leading to their increased use as “standard” catalysts (Formenti et al., 2018; Pal and Pal, 2025). The Cu-PANI-AC-A electrocatalyst outperformed previous systems and provides a sustainable method for electrochemical aniline production by enabling effective nitrobenzene reduction to aniline with 82% selectivity. It has potential for fuel cell cogeneration is typically used for heat and power due to its stability and efficacy (Daems et al., 2018; Khanra et al., 2025). Using cheap Ni/Co nanoparticle catalysts with hydrazine hydrate, provides room-temperature, aqueous-phase chemo selective reduction of nitro compounds that achieves high conversions and selectivity (Rai et al., 2014; Zhao et al., 2024). This review found important structure–activity connections, mechanistic pathways and degradation mechanisms that control catalyst performance by contrasting noble metals (such as Pt, Pd and Au) with non-noble substitutes (such as Cu, Fe, Co and Ni-based materials). The catalyst, which is frequently combined with protons from water (H_2_O) as the hydrogen source, acts as a conductive platform in electrocatalytic systems to enable direct electron transfer from the electrode to the substrate (Hasan et al., 2024; Islam et al., 2024). With their easy recovery, resistance to leaching and deactivation and ability to activate substrates through non-covalent interactions, (Electrostatic interaction for parallel structure with hydrogen bonding, π–π* stacking), metal nanoparticles supported on graphene oxide (GO) have become very promising nanocatalysts in the last decade. Compared to their monometallic counterparts or the bare support, bimetallic and trimetallic nanoparticles on GO display remarkable synergistic features (Hoseini Chopani et al., 2020; Kumar et al., 2023). Bimetallic Supports mediates electronic synergy; promotes alloy formation (Hoseini Chopani et al., 2020)]. A crucial industrial transition, sustainable nitroarene reduction has been achieved using magnetically recyclable nanocatalysts. In addition to improving durability, catalytic activity and simple magnetic separation, incorporating design strategies that prevent aggregation (Shokouhimehr, 2015; Zarei et al., 2024). The selective reduction of electron-rich nitro heteroarenes is difficult but essential transformation for the synthesis of bioactive molecules. Assessed contemporary catalytic techniques (metal-free systems, hydrogenation and transfer hydrogenation) (Rostami et al., 2024; Vengatesh and Nanjan, 2022). The application of bulk and supported non-noble metal catalysts for the liquid-phase hydrogenation of bi- and polyfunctional organic molecules in flow mode is covered in a recent study. When other substituents are present, the primary focus is on the selective reduction of one functional group (NO_2_, C
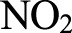
C, C
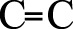
N, C
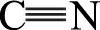
O and C
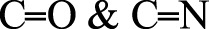
N) (Nuzhdin et al., 2024; Tang et al., 2024) (Scheme 1).

The two main types of catalysts are non-noble metal catalysts (Fe, Ni, Cu, Co, Mo and Mn) and noble metal catalysts (Pt, Au, Pd, Ag, Rh and Ru).

### 1.1 Noble metal catalyst

Noble metal catalysts, including platinum (Pt), silver (Ag), palladium (Pd), gold (Au), rhodium (Rh) and ruthenium (Ru), are widely known for their remarkable catalytic properties in a variety of chemical reactions. Because of their high activity, superior thermal stability and potent oxidation and corrosion resistance, these metals are essential for use in both industrial and environmental settings. Their distinct surface characteristics and electrical structure allow for effective reactant activation and adsorption, which improves catalytic performance. Silver (Ag) nanoparticles’ plasmonic properties play a key role for the catalytic conversion of nitrobenzene (Ph-NO_2_) to aniline (Ph-NH_2_). When Ag nanoparticles are exposed to light, generate hot electrons *via* localized surface plasmon resonance (LSPR), which are then transported to the nitrobenzene molecules absorbed on the catalytic surface. These energetic electrons help intermediary species like nitroso benzene (Ph-NO) and phenylhydroxylamine (Ph-NHOH) facilitate the nitro (–NO_2_) group gradually into an amino (–NH_2_) group (Guo et al., 2023; Park et al., 2024). In an “efficient, environmentally friendly” method of producing Pt nanoparticles in water, gum acacia has been employed as a stabilizing and reducing agent. The hydrogenation of nitroarenes to arylamines is efficiently catalyzed by colloidal platinum using molecular hydrogen as the reductant. Gum acacia has been used as a stabilizing and reducing agent in the efficient environmentally friendly synthesis of Pt nanoparticles from H_2_PtCl_6_.6H_2_O in water. Excellent product yields were obtained with a variety of substrates and the catalyst remained reliable across several cycles (Sreedhar et al., 2011; Gogoi et al.). By combining the hydrogenation of nitro groups with the dehydrogenation of ammonia borane (NH_3_BH_3_), the Pd@MIL-101 catalyst transforms nitrobenzene into aniline. In a methanol/water solution, tiny Pd nanoparticles (∼2.5 nm) confined with the pores of MIL-101 promote the quick release of H_2_ from NH_3_BH_3_ (Aprile et al., 2024; Yang Q. et al., 2015). Nitrobenzene at room temperature is rapidly reduced by the *in situ* produced hydrogen, completing the reaction in just 1.5 min with a yield of >99% (Campbell et al., 2024; Singha Hazari et al., 2024). Ru nanoparticles are stabilized by a polymer-immobilized ionic liquid coated with phosphorus oxide (RuNP@O=PPh_2_)-PEGPIILS) catalyzes the reduction of nitrobenzene to aniline in two steps. Under mild conditions, nitrobenzene is first partially reduced to N-phenylhydroxylamine using hydrazine hydrate as the reducing agent. With excellent selectivity, the reaction takes place in ethanol between 25 °C and 40 °C. The N-phenylhydroxylamine intermediate then undergoes further reduce to aniline at elevated temperature (60 °C) or longer reaction times (Paterson et al., 2023; Stergiou et al., 2022). Using RuNP@O=PPh_2_-PEGPIILS catalysts at 25 °C, Paterson et al. extracted NPhOH with >99% yield (Paterson et al., 2022; Paterson et al., 2023). NPhOH was identified by Doherty et al. as the main intermediate in Au/PIILs-catalyzed reduction, and its stability was determined by the solvent composition (Doherty et al., 2025). Gold nanoparticles (AuNPs) supported by phosphine oxide-decorated polymer-immobilized ionic liquids (PIILs) catalyze the multi-step reduction of nitrobenzene to aniline. The primary intermediate that was observed is N-phenylhydroxylamine, which is first partly reduced from nitrobenzene. At low water content, the reaction proceeds quickly. When N-phenylhydroxylamine is further reduced, aniline is the result. Ethanol encourages the synthesis of azoxybenzene whereas water favors the formation of N-phenylhydroxylamine (C_6_H_5_NHOH). The solvent is crucial. The choice of reducing agent such as Dimethylamine borane (DMAB) or NaBH_4_ has a major effect on the reaction rate; DMAB exhibits higher activity (Doherty et al., 2025). The catalyst discussed in the article is a rutile-type TiO_2_ photocatalyst which is formed into tiny TiO_2_-zircon bead composites (referred to as TiO_2_-ZB) and is commercially available as MT-150A from TAYCA Corporation, Osaka, Japan. With an apparent quantum efficiency of 84% in the isopropanol-nitrobenzene (IPA-NB) reduction process, this form of rutile TiO_2_ is notable for its high effectiveness. Using IPA as a sacrificial hydrogen donor, the TiO_2_-ZB catalyst is highly effective in photocatalytically hydrogenating nitrobenzene to aminobenzenes when exposed to UV light from black light. By optimizing reactant-catalyst contact and TiO_2_ activation the spiral flow reactor design improves performance (Kominami et al., 2025).

### 1.2 Non-noble metal catalyst

Non-noble metal catalysts have attracted a lot of attention as affordable and eco-friendly substitutes for expensive noble metal-based catalysts (such as Pt, Pd, Rh, *etc.*). These catalysts are mostly composed of transition metals (Fe, Co, Ni, Cu and Mn) and their oxides, carbides, nitrides and phosphides, as well as carbon-based materials like graphene and doped nanocarbons. Compared to noble metals, non-noble metal catalysts are more accessible, more affordable and can be structurally and compositionally turned for specific applications. One of the most widely used catalytic processes in the bulk and fine chemical industries is the reduction of nitro compounds to their corresponding amines. These catalysts are commonly utilized as “standard” catalysts because of the impressive results of recent catalyst advances using inexpensive metals like Fe, Co, Ni and Cu (Zirak et al., 2025). A simple technique was developed to produce Ni-Sn bimetallic nanoparticles with a controlled size and composition under ambient conditions using inexpensive metal salts. By altering the stoichiometric ratio of Ni and Sn precursors, different chemical compositions of Ni_100_, Ni_74_Sn_26_, Ni_59_Sn_41_ and Ni_50_Sn_50_ nanoparticles were formed. The nanoparticles have been described using X-ray diffraction (XRD) (which revealed alloy phases like Ni_3_Sn and Ni_3_Sn_2_), Transmission electron microscope (TEM) (which showed irregular/spherical forms with diameter of 4.5–13 nm), HR-TEM, and energy dispersive X-ray (EDX) (which verified composition) (Shah et al., 2015). Cu@C a new Cu-based MOF precursor calcined at 700 °C has remarkable catalytic activity when nitrobenzene is reduced to aniline with NaBH_4_. Many previously reported non-noble metal catalysts, achieving 100% conversion in just 8 min. After acid etching and several reuse cycles without reactivation it maintained over 90% efficiency, demonstrating exceptional stability. Characterization tests confirmed the material’s high surface area and uniform distribution of Cu nanoparticles. It highlights Cu@C as a low-cost, efficient replacement for noble metal catalysts in the reduction of nitro compounds, with potential applications in wastewater treatment and green chemistry (Tavakolian et al., 2025). A. Mahajan and M. Gupta developed a recyclable nanocatalyst hybrid ceria or iron-doped-cerium oxide (Fe@CeO_2_) which enables both the production of benzopyran derivatives in eco-friendly solvents and the hydrogenation of nitroarenes to anilines. Numerous methods were used to characterize the catalyst, which showed excellent stability, a high surface area and photoluminescent qualities. In mild conditions it produced high yields (up to 95%) in the synthesis of benzopyran and the reduction of nitroarene. Over the course of five successive runs the catalyst’s efficiency and recyclability were demonstrated. It highlights the catalyst’s potential for environmentally friendly and sustainable chemicals (Mahajan and Gupta, 2021).

Critical gaps in nitroarene reduction remain unfilled despite multiple reviews: (1) Without a direct, side-by-side kinetic comparison (e.g., does Cu@C really replace Pd/C at scale?), most assessments concentrate on either noble or non-noble metals. (2) Although the significance of the hydrogen source is sometimes considered secondary, our examination of Table 1 shows that the donor selection (NaBH_4_ vs. H_2_ vs. N_2_H_4_) has a greater influence on the reaction process than the metal itself. (3) It is uncommon to quantify the stability-reusability dilemma (high initial activity vs. fast deactivation). Thus, this work is novel in the following ways; (i) creating the first complete performance matrix (Table 2 and 3) that cross-references metal type, support, hydrogen source, time and yield under standard conditions; (ii) determining that the current “unnoticed champions” with turnover frequencies TOFs exceeding 100 h^-1^ are bimetallic Ni-Sn and defect-engineered carbon catalysts; and (iii) putting forth a tree of choices model for catalyst selection based on industrial vs. environmental applications.

**TABLE 1:** Various hydrogen sources for conversion of nitroarenes into amino arenes.

SNO	Hydrogen source	Reactant	Corresponding catalyst	Product	Selectivity	Yield	References
1	H_2_ (*via* NaBH_4_) NaBH_4_+PMS	Nitrobenzene	Co (SA + NPs)-C/N (Co single atoms + nanoparticles on N-doped carbon) TiO2/ZnO-chitosan gel beads Cu@C (from Cu-MOF) Ni74Sn26 nanoparticles Ni (PPh_3_)_4_	Aniline	>99% >98% >99% >99% >90% >95%	100% 100% 100% 100% 96.6% 94%	Qi et al. (2024) Anusuya et al. (2021) Tang et al. (2023) Shah et al. (2015) Zhou et al. (2025) Akbarizad et al. (2024)
2	Ethylene glycol/KOH	Nitrobenzene	CuNPs/Celite (5% w/w)	Aniline	>99%	>99%	Martina et al. (2023)
3	Hydrazine hydrate	Nitrobenzene	CuNCNT (N-doped Cu) Cu@porous carbon Co_3_O_4_ nanoparticle	Aniline	>95% >99% >95%	97% 99% 99%	Zhang et al. (2023a) Zou et al. (2020) Patil et al. (2025)
4	Glycerol	Nitrobenzene	Cu-based catalyst	Aniline	92% (aniline	100%	Moran et al. (2020a)
5	H_2_ (atmospheric)	Nitrobenzene and functionalized nitroaromatics.	TiO_2_@N-AC NiFe hydrogenase on carbon black (biocatalyst)	Aniline and various substituted or heteroaromatic amines.	∼96% (for derivatives) 99%	100% 100%	Sokolova et al. (2024) Guo et al. (2023)

**TABLE 2:** Noble metal nanocatalysts for nitroarene reduction.

S no	Reactant	Reducing agent	Noble metal catalysts	Product	Yield (%)	Time	Reaction temperature and pressure	References
1	Nitrobenzene	H_2_ (via NaBH_4_)	Pd@MIL-101	Aniline	>99	1.5 min	RT, ambient	Campbell et al. (2024), Yang et al. (2015a)
2	Nitrobenzene	H_2_ (gas)	Pt NPs/Gum acacia	Aniline	>99	3 h	RT, 1 atm H_2_	Sreedhar et al. (2011)
3	Nitrobenzene	N_2_H_4_.H_2_O	RuNP@O=PPh2-PEGPIILS	N-phenylhydroxylamine to aniline	>99	2 h	25 °C–60 °C, inert	Paterson et al. (2023)
4	Nitrobenzene	DMAB or NaBH_4_	AuNPs/PIILs	Aniline	>99	45 min	RT, ambient	Doherty et al. (2025)
5	Nitrobenzene	Isopropanol (photocatalytic)	TiO_2_-ZB (rutile TiO_2_)	Aniline	High	Continuous flow	UV light, RT	Kominami et al. (2025)
6	4-Nitrophenol	NaBH_4_	Pt NPs/viologen micelles	4-Aminophenol	93	3.5 h	RT, ambient	Kantam et al. (2012)
7	Nitrobenzene	H_2_ (gas)	Pt/PSP (hyper-crosslinked polystyrene)	Aniline	>99	2 h	40 °C, 5 bar H_2_	Cárdenas-Lizana et al. (2013)
8	Nitrobenzene	H_2_ (gas)	Pd/Fe_3_O_4_ NPs	Aniline	>95	70 min	RT, 1 atm H_2_	Zhang et al. (2011b)
9	Nitrobenzene	Hydrazine hydrate	Pd/C	Aniline	95	5 min	80 °C, ambient	Li et al. (2014)
10	Nitrobenzene	H_2_ (gas)	Ag/CeO_2_ core–shell	Aniline	>95	4 h	80 °C, 1 atm H_2_	Shimizu et al. (2010)
11	Nitrobenzene	H_2_ (gas)	Ag/Al_2_O_3_	Aniline	>99	3 h	100 °C, 1 atm H_2_	Qusti et al. (2014)
12	Nitrobenzene	NaBH_4_	Ag/Fe_3_O_4_–SiO_2_	Aniline	>95	8 min	RT, ambient	Liu et al. (2021a)
13	Nitrobenzene	H_2_ (gas)	Ag@SiO_2_ core–shell	Aniline	>99	5 h	120 °C, 10 bar H_2_	Patzsch et al. (2019)
14	Nitrobenzene	NaBH_4_	Ag/GQDs	Aniline	>99	6 min	RT, ambient	Liu et al. (2021a)
15	Nitrobenzene	Formic acid	Rh–terpyridine complex	Aniline	>99	12 h	60 °C, water	Tan et al. (2021)
16	Nitrobenzene	Hydrazine hydrate	Rh NPs/support	Aniline	>99	1.5 h	60 °C, ambient	Liu et al. (2022a)
17	Nitrobenzene	H_2_ (gas)	RuNi single-atom alloy	Aniline	>99	3 h	80 °C, 5 bar H_2_	Liao et al. (2023)
18	Nitrobenzene	H_2_ (gas)	Ru/CNT	Aniline	>99	4 h	80 °C, 10 bar H_2_	Li et al. (2025b)
19	Nitrobenzene	H_2_ (gas)	Pt–Ga dual-atom/N-graphene	Aniline	>99	6 h (electrolysis)	RT, 0.1 V vs. RHE	Liu et al. (2025)
20	Nitrobenzene	NaBH_4_	PdO/TiO_2_	Aniline	>99	12 min	RT, ambient	Lu et al. (2025)
21	Nitrobenzene	H_2_ (gas)	PdC_x_ nano cubes	Aniline	>99	2 h	50 °C, 2 bar H_2_	Yamaguchi et al. (2025)

**TABLE 3:** Non-noble metal nanocatalysts for nitroarene reduction.

S NO	Reactant	Reducing agent	Non noble catalysts	Product	Yield (%)	Time	Reaction temperature and pressure	References
1.	Nitroarene	NaBH_4_	TiO_2_/ZnO-chitosan gel beads	Aniline	100	0.15 min	45 °C ambient pressure	Anusuya et al. (2021)
2	Nitroarene	NaBH_4_	Cu@C (from Cu-MOF)	Aniline	100	8 min	RT, ambient pressure	(Tang et al., 2023)
3	Nitroarene	NaBH_4_+ PMS	Fe-N/BC biochar	Aniline	96.6	15 min	RT, ambient pressure	Zhou et al. (2025)
4	Nitroarene	NaBH_4_	Ni (PPh_3_)_4_	Aniline	94	20 min	RT, ambient pressure	Akbarizad et al. (2024)
5	Nitroarene	NaBH_4_	Ni_74_Sn_26_ nanoparticles	1,2-Benzenediamine	100	2.5 min	RT, ambient pressure	Shah et al. (2015)
6	Nitroarene	Na_2_Se	Se/NaBH_4_/NaOH	Aryl amines	94	2 h	100 °C, ambient pressure	Capperucci et al. (2023)
7	Nitroarene	NaBH_4_	NaBH_4_/CuCl_2_	Phenethylamines	83–97	10–30 min	RT, ambient pressure	d'Andrea and Kristensen (2023)
8	Nitroarene	NaBH_4_	Co-pol (N-doped resin)	Aniline	90	15 min	RT, ambient pressure	Petrelli et al. (2023)
9	Nitroarene	NaBH_4_	Ni@Fe-CeO_2_chitosan	Aniline	95	15 min	50 °C, ambient pressure	Mahajan and Gupta (2021)
10	Nitroarene	H_2_ (*in situ*)	RuNP@O=PPh_2_-PEGPIILS	N-Arylhydroxylamine	>99	2 h	25 °C, inert atmosphere	Paterson et al. (2022)
11	Nitrobenzene	Bi_2_MoO_6_’Methanol, water	Bi_2_MoO_6_	Aniline	>99	12–24 h	ambient	Xie et al. (2019)
12	Nitrobenzene	H_2_	TiO_2_@N-AC	Aniline	>99	12–24 h	25 °C, low pressure	Guo et al. (2023)
13	Nitrobenzene	Hydrazine hydrate/Co (II) catalyst	Co (II) catalyst	Aniline	99	20 min	Room temperature, Atmospheric pressure	Manikandan et al. (2025)
14	Nitrobenzene	Hydrazine hydrate	CuNCNT (N-doped Cu)	Aniline	97	1 h	28^O^C Atmospheric	Zhang et al. (2023a)
15	Nitrobenzene	Hydrazine hydrate	Ru/MIL-101	Aniline	99	1 h	100 °C Atmospheric	Fang et al. (2022)
16	Nitrobenzene	Hydrazine hydrate	Cuprous carbon	Aniline	99	1.5 h	85 °C Atmospheric	Zou et al. (2020)
17	Nitrobenzene	Hydrazine hydrate	Co_3_O_4_ nanoparticle	Aniline	99	1 h	Room temperature, Atmospheric pressure	Patil et al. (2025)
18	Nitrobenzene	Hydrazine hydrate	Fe-doped TiO_2_	Aniline	96	2 h	100 °C Atmospheric	Ji et al. (2019)
19	Nitrobenzene	Ethylene glycol/KOH	CuNPs/Celite (5% w/w)	Aniline	>99	∼9 min	130 °C Atmospheric	Martina et al. (2023)
20	Nitrobenzene	Hydrazine hydrate	FeCl_3_·6H_2_O/2,2′-Bipyridyl	Aniline	92	1 h	100 °C Atmospheric	Hung et al. (2022)
21	Nitrobenzene	H_2_ (*in situ* from NaBH_4_)	Co (SA + NPs)-C/N (Co single atoms + nanoparticles on N-doped carbon)	Aniline	>99	2.5 min	Room temp (25 °C)/Ambient (1 atm)	(Qi et al., 2024)

### 1.3 Hydrogen sources for reduction

The nitroarene hydrogenation mechanism is extremely sensitive to the conditions of the reaction. Selectivity is greatly influenced by the polarity and acidity of the solvent; for example, ethanol favors the synthesis of azoxybenzene over Au catalysts, whereas water promotes the formation of N-phenylhydroxylamine (Doherty et al., 2025). The depth of the reaction is determined by temperature; at higher temperatures (e.g., 60 °C), intermediates such as N-phenylhydroxylamine can be completely reduced to aniline over Ru catalysts, whereas at lower temperatures (25 °C–40 °C), the partial reduction product can be isolated (Paterson et al., 2023; Stergiou et al., 2022). The mechanism is also controlled by the hydrogen source: transfer hydrogenation donors such as glycerol proceed *via* different dehydrogenation-coupled pathways (Sun et al., 2010), whereas molecular H_2_ requires activation on metal sites. Surface functional groups enable hydride transfer; defect sites anchor active species for nitroarene reduction (Liao et al., 2023; Qi et al., 2024). NaBH_4_ facilitates quick hydride transfer for fast kinetics (Shah et al., 2015; Tang et al., 2023). A variety of hydrogen sources have been used, including different catalytic systems like electrochemical or photocatalytic processes, transfer hydrogenation donors and molecular hydrogen gas. Electrochemically active microorganisms facilitate electron transport at the biocathode should be the microbial fuel cell. These electrons convert nitrobenzene to aniline by combining with protons in the solution to form active hydrogen species (Wang et al., 2011). In photocatalytic systems, molecular hydrogen is commonly employed as a direct hydrogen source. Photocatalysts, such as Titanium dioxide (TiO_2)_, CdS or metal-decorated semiconductors (e.g., Pt/g-C_3_N_4_, Cu/TiO_2_) absorb light to generate electron-hole pairs. The electrons reduce H_2_ to form reactive hydrogen species (e.g., H• or H^−^) on the catalyst surface, which then reduce nitrobenzene to aniline (Guo et al., 2023). Glycerol acts as a renewable and environmentally friendly hydrogen donor during the transfer hydrogenation process. Glycerol acts as a renewable hydrogen donor, being oxidized to products such as dihydroxyacetone or glyceraldehyde, while simultaneously providing hydrogen equivalents that reduce the nitro group (-NO_2_) of nitrobenzene to the amino group (-NH_2_) of aniline (Moran et al., 2020a). The reaction system uses molecular hydrogen directly at 333 K (60 °C) and 4 MPa of pressure. The (-NO_2_) of nitrobenzene is reduced to the (-NH_2_) of aniline by the dissociation of H_2_ into reactive hydrogen species (such as atomic hydrogen or hydrides) made possible by ultrafine platinum nanoparticles (PtNPs) supported on multi-walled carbon nanotubes (MWNTs) (Sun et al., 2010).

### 1.4 Catalytic reduction of nitro compounds by various catalysts

#### 1.4.1 Platinum-based catalysts

The metal oxidation, dehydrogenation, and hydrogen capacity of supercapacitors in batteries and sensors are all commonly facilitated by Pt-based nanoparticles (Pt NPs) (Aday et al., 2016; Feng et al., 2024; Gawande et al., 2012; Soni et al., 2025). Furthermore, these nanomaterials are well-known and have been among the most popular catalysts for reduction reactions and heterogeneous catalysts (Lara and Philippot, 2014; Nie et al., 2012; Rohmah et al., 2025). Compared to catalysts based on other metals such as Au, Ag, Ni, Ir, Rh and Ru, platinum-based catalysts are typically faster and more dynamic. A commercial Pt/C catalyst, for example, to reduce nitro compounds containing bromine or chlorine. In the redox processes of a hydrogen molecule exchange, the catalysts are based on the Pt. One example is the reduction of nitro-compounds containing chlorine or bromine using a commercial Pt/C catalyst. The reactions produced a yield of more than 99 percent in less than 5 minutes when 1,4-cyclohexadiene was used as an H_2_ source (Jin et al., 2021; Rueping et al., 2011).

**SCHEME 2:**
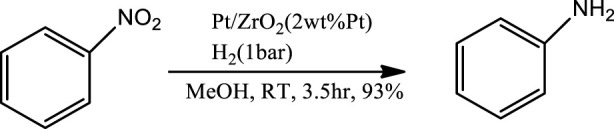
Conversion of nitrobenzene into Aniline catalyzed by Pt/ZrO_2_.

N-heterocyclic carbene (NHC)-stabilized platinum nanoparticles (Pt NPs) were produced by decomposing [Pt(dba)_2_] with carbene ligands in tetrahydrofuran (THF) at room temperature under 3 bar hydrogen. These Pt@NHC catalysts demonstrated high activity by efficiently hydrogenating p-substituted nitroarenes. NHC ligands increase the stability of the catalyst and stop unintended agglomeration. According to the study, catalysts can tolerate functional groups including carbonyl, hydroxyl and benzyloxy (He et al., 2019; Lara et al., 2014; Nie et al., 2020) (Scheme 2).

**SCHEME 3:**
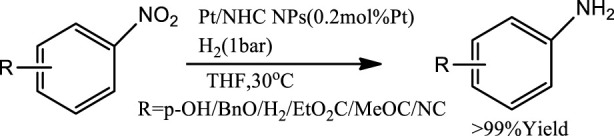
Conversion of nitrobenzene into aniline by using Pt/NHC NPs.

Platinum nanoparticles (Pt NPs) stabilized on carbon, Al_2_O_3_ and hyper-crosslinked polystyrene (PSP) were used as catalysts to hydrogenate p-substituted nitroarenes (substituents: OCH_3_, -H, -Br, -Cl, -COOH, -NO_2_) producing >99% amine yields and TOFs up to 61s^-1^. In the liquid phase, these catalysts successfully reduced p-chloronitrobenzene. Metal leaching is avoided and mechanical stability is improved by the hyper-crosslinked polystyrene support. Both effective and selective hydrogenation are achieved by the Pt/PSP structure. The catalysts’ performance and stability indicate that they have a significant potential for real-world use (Cárdenas-Lizana et al., 2013). To effectively hydrogenate the nitro group in nitrobenzene, platinum (Pt) serves as the main active site for splitting molecular H_2_ into reactive atomic hydrogen. By selectively converting–NO_2_ to–NH_2_ while limiting aromatic ring hydrogenation, it offers high selectivity toward aniline. Compared to metals like Ni, Pt performs well under mild conditions and at lower temperatures and pressures. Adsorption determines how water affects Pt: liquid H_2_O limits the process by competitively blocking active sites, whereas vapor H_2_O speeds it up (Zhou et al., 2024). The reduction of p-nitrophenol to aniline was catalyzed by platinum nanoparticles supported in bridged micelles (1,1′-dioctadecyl-4,4′-bipyridinium bromide) using NaBH_4_ in a DMF-water mixture. The micelles’ core-shell structure of pyridine and viologen cations allowed for effective catalysis. In 3.5 h, a 93% aniline yield was obtained in methanol. This method demonstrates an advanced technique for halonitrobenzene reduction while maintaining halogen substituents (Campos et al., 2022; Kantam et al., 2012; Luo et al., 2023) (Scheme 3).

To improve the electronic structure of the active sites, Pt-M (p-block metal) dual-atom catalysts, especially Pt-Ga supported on N-doped graphene, regulate p-d orbital hybridization. By adjusting the adsorption energies of hydrogen species and nitrobenzene intermediates, this hybridization lowers the energy barriers for stepwise electrocatalytic hydrogenation and suppresses the competing hydrogen evolution process (HER). Because of this, the catalyst performs exceptionally well in the nitrobenzene reduction reaction (NBRR) under mild electrochemical conditions and exhibits remarkable activity and high selectivity toward aniline synthesis (Liu et al., 2025).

#### 1.4.2 Gold-based catalysts

**SCHEME 4:**
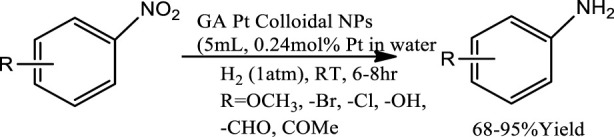
Hydrogenation of nitroarenes catalyzed by GA-Pt NPs (Sreedhar et al., 2011).

Calorimetric sensors commonly use gold nanoparticles, tactile frameworks, nano chemistry and nanomaterials science (Doherty et al., 2025; Rohmah et al., 2025; Su and Kanjanawarut, 2009; Xu et al., 2012). Up until recently, gold was thought to have poor catalytic activity. It was observed that the chemo selective hydrogenation of nitroarenes was carried out by calcium alginate gel (Saha et al., 2010), TiO_2_, Fe_2_O_3_ (Corma and Serna, 2006), hydrogenation of graphene oxide (Choi et al., 2011), tannic corrosive graphene oxide functionalized (Zhang Y. et al., 2011), filaments of polyaniline (Han et al., 2010), small scale organized paper network (Koga and Kitaoka, 2011) and alginate gel of calcium (Saha et al., 2010) supported gold nanoparticles counting various useful groups (Mitsudome and Kaneda, 2013). HAuCl_4_ and commercially available NAP-MgO undergo an ion-exchange process to produce gold nanoparticles supported on magnesium oxide (NAP-MgO). In this way gold was applied to the surface of NAP-MgO with gold in the zero-valent state as a heterogeneous catalyst. The estimated size of nanoparticles is between 5 and 7 nm (Sreedhar et al., 2011) (Scheme 4).

#### 1.4.3 Catalysts based on palladium

Since catalysts have a stronger catalytic effect, they have been widely studied for the exchange of hydrogenation reactions. Using palladium-based heterogeneous catalysts, significant advancements in the chemo selective hydrogenation of nitroarenes have recently been made (Li et al., 2011; Rubio-Marqués et al., 2013). The catalyst, usually Pd, Ni or Pt-based (e.g., Pd/Al_2_O_3_ in related studies) is essential for hydrogenating nitrobenzene to aniline by reducing the activation energy. This enables the reaction to proceed efficiently at moderate pressures and temperatures. It facilitates the adsorption of hydrogen gas (H_2_) on its surface, generating active hydrogen species that progressively reduce the nitro group (-NO_2_) *via* intermediates such as nitrosobenzene and phenylhydroxylamine, ultimately forming C_6_H_5_NH_2_ with superior selectivity (Rohmah et al., 2025). Palladium nanoparticles (PdNPs) particularly those produced by solution phase deposition (small NPs ∼5 nm) coated on polymer-based spherical activated carbon (PBSAC) function as highly active heterogeneous catalysts for the hydrogenation of nitrobenzene to aniline using H_2_ gas (Fu et al., 2023). For example, Pd/Fe_3_O_4_ NPs that are easily magnetically separable were created to reduce a number of nitro compounds in ethyl alcohol in 60–75 min with excellent yields in a hydrogen environment (Zhang F. et al., 2011; Zhang et al., 2018). The attractive Pd/Fe_3_O_4_ catalyst was primarily created to reduce nitroarenes with carbonyl groups in a selective manner (Boyall et al., 2024; Hasan et al., 2023). A new method for the reduction of nitro compounds catalyzed by Pd/C was developed, using 1,4-cyclohexadiene as hydrogen source. This method is quick, safe and efficient.

**SCHEME 5:**
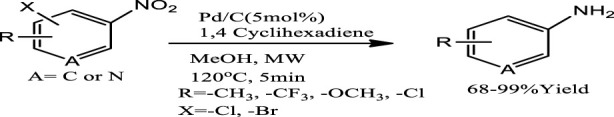
In the presence of Pd/C, the reduction of aromatic nitro compounds was aided by microwaves (MW).

**SCHEME 6:**
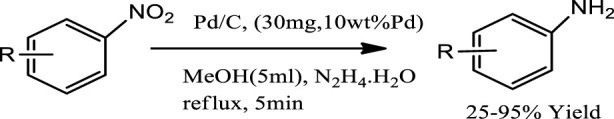
Hydrogenation of nitro compounds catalyzed by Pd/C and N_2_H_4_.H_2_O.

Hydrazine monohydrate (N_2_H_4_.H_2_O), a well-known hydrogen source was reacted with a Pd/C catalyst to produce aromatic amines. Within 5 minutes, the conversion in methanol at 80 °C changed from 25% to 95%. The Pd/C catalyst can be operated up to five times without experiencing any serious deactivation (Herrera et al., 2025; Li et al., 2014) (Scheme 5,6).

**SCHEME 7:**
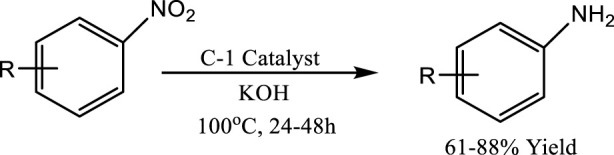
C-1 catalyzed selective reduction of various nitroarenes.

The most prevalent element in the universe is elemental carbon which comes in a number of different forms such as diamond, graphite and amorphous carbon (Yang H. et al., 2015). It is a useful component for a variety of applications such as catalyst supports (Trasarti et al., 2014), fuel cell (Stiles, 1987) and other applications. For many years the catalytic hydrogenation of nitro compounds has been dominated by metal catalysis. Despite their increased reusability, heterogeneous catalytic systems are costly and environmentally harmful. More recently, successful development of metal-free catalysts for H_2_ enactment has been studied (Zheng and You, 2012). Preparation of aniline is more economical, safe and environmentally beneficial. According to Bao et al., reduced graphene oxide (rGO) can be used effectively as a catalyst to reduce nitroarenes without metals. Up to 97% of the reactions were completed in 4–9 h. Hydrazine monohydrate was utilized as hydrogen source and solvent. C-0 stands for carbon compounds that were not subjected to base treatment. Using different bases (KOH, NaOH, K_2_CO_3_ and Na_2_CO_3_, respectively) and a set ratio of wet Resorsinol-Formaldehyde (RF) gel to base (i.e., 1:1), C-1, C-2, C-3 and C-4 were made. When isopropanol was used as a soluble hydrogen source, the C-1 catalyst was used for the reduction reaction to generate aniline derivatives in the presence of KOH. Within 24–48 h the reaction progressed with desirable yields under high pressure and temperature. The usefulness of the additional reducible substituents that were attached to the aromatic ring was maintained (Yang H. et al., 2015) (Scheme 7).

**SCHEME 8:**
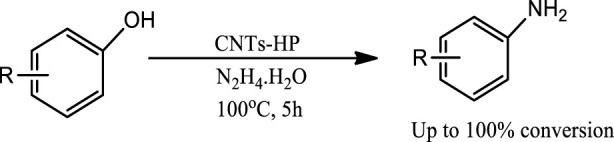
CNTs-HP catalyzed various nitroarenes.

Functionalized carbon nanotubes (CNTs) are now commonly used as catalysts in a variety of chemical processes (Fujita et al., 2014; Wu et al., 2015). Su et al. created a novel hydrogen peroxide functionalized carbon nanotube (CNT-HP). CNT-HP is green catalyst that provides a particular reduction of nitroarenes with high conversion and high yields by using hydrazine monohydrate as a hydrogen donor. CNTs were treated with 30% H_2_O_2_ at different temperatures to prepare a more active catalyst. CNT-HP60 was reported as an effective catalyst for the hydrogenation of nitro compounds. CNT-HP was compared to nitric acid oxidized carbon nanotubes (CNTs) for reduction. They reported that in CNT-HP, the conversion of nitroarenes to aniline derivatives is more efficient (Wu et al., 2015) (Scheme 8).

**SCHEME 9:**
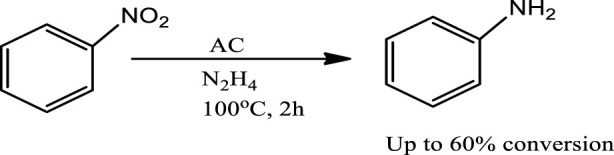
AC catalyzed reduction of nitrobenzene.

Using hydrogen peroxide and ammonium salts, the surface functionalized (AC) catalyst was appropriately doped with nitrogen and oxygen. It was reported that nitrogen and oxygen dopants changed the surface structure of AC, increasing its catalytic activity for nitrobenzene hydrogenation. Additionally, hydrazine hydrate was employed as a source of hydrogen donor (Fujita et al., 2014) (Scheme 9).

Using NaBH_4_ as the hydrogen donor, the PdO/TiO_2_ catalyst allows for the very effective catalytic transfer hydrogenation of nitrobenzene to aniline, where PdO nanoparticles supported on TiO_2_ first activate the nitro group and facilitate hydrogen generation from NaBH_4_ (Lu et al., 2025). When compared to pure Pd nanocubes, the palladium carbide nanocube catalyst (PdC_x_ NCs) significantly enhances the reduction of nitrobenzene to aniline by improving H_2_ activation and nitrobenzene adsorption, yielding a turnover frequency (TOF) that is 39 times greater (Yamaguchi et al., 2025).

#### 1.4.4 Silver-based catalysts

**SCHEME 10:**
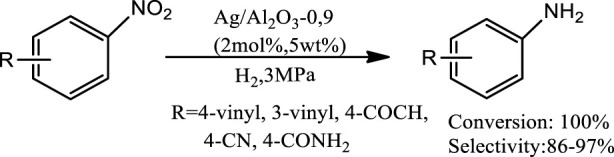
Conversion of nitroarenes into amino arenes in the presence of Ag/Al_2_O_3_ (Shimizu et al., 2010).

In research, silver-based catalysts are often used in oxidation reactions (Guo et al., 2023; Taheri-Ledari et al., 2020). By stabilizing important intermediates (nitroso and hydroxylamine species), Ag NPs facilitate the selective hydrogenation of the–NO_2_ group to–NH_2_, producing high aniline yields with few byproducts (Kumar et al., 2025). On a large scale, the remarkable focus on Ag catalysts for reduction reactions, which is currently uncommon, is utilized to lower the activation barrier of the reaction (Corma and Garcia, 2008; Davis et al., 2013). The active site for low-barrier B-H activation from NaBH_4_ is electron-deficient single-atom silver (AgSA) on γ-Al_2_O_3_, which facilitates the easy production of silver-hydride species that are essential for the hydrogenation of nitrobenzene to aniline (Li H. et al., 2025). They can be used to reduce 4-nitrophenol and 4-aminophenol efficiently in a short time. For instance, Kaneda et al. used Ag/CeO_2_ NPs to chemoselectively reduce the range of nitro-compounds in a hydrogen medium with an efficiency of >95% was synthesized. The carbonyl groups were protected and a high yield of the required compounds was achieved in a selective manner. MgO, CeO_2_, ZrO_2_, TiO_2_, SnO_2_ and Al_2_O_3_ were among the materials utilized to construct the catalytic supports; it was discovered that Al_2_O_3_ had the greatest surface area (Qusti et al., 2014; Shimizu et al., 2010) (Scheme 10). The primary active sites that dissociate and activate molecular H_2_ are silver nanoparticles (Ag NPs) which are distributed on a sustainable hybrid silica/carbon substrate made from rice husk waste. This makes it possible to transfer hydrogen to the nitro group of nitrobenzene in a selective and effective manner, resulting in a high conversion to aniline with outstanding chemo selectivity in mild conditions (Unglaube et al., 2021). The main active sites in the curcumin/melamine-functionalized magnetic nanocatalyst are silver (Ag) nanoparticles that facilitate electron transfer and hydride donation from the reducing agent to reduce the nitro group (-NO_2_) of nitroarenes (Khaleghi et al., 2023; Zhang et al., 2024). Specifically, compared to vinyl ether, nitrile and amide linkages, the Ag/Al_2_O_3_ NPs are very selective for the nitro group reduction. High yields and selectivity of the products were obtained. The catalysts were created using support materials like MgO, CeO_2_, ZrO_2_, TiO_2_ SnO_2_, and Al_2_O_3_. Al_2_O_3_ has been shown to have the maximum surface area.

In the presence of NaBH_4_, 4-nitrophenol was reduced to 4-aminophenol using the Fe_3_O_4_/SiO_2_ supported silver catalyst, which was made as magnetic nanoparticles using silver nitrate (AgNO_3_) as a silver source.

The Ag nanoparticle core (reduced from AgNO_3_, using aldehydes, with smaller sizes from stronger reducers like CH_2_O) acts as the main active site for activating and dissociating H_2_ gas, enabling the reduction of the nitro group. Nitrobenzene can be hydrogenated to aniline using the mesoporous core-shell Ag@SiO_2_ nanospheres as an efficient heterogeneous catalyst (Patzsch et al., 2019; Zhao et al., 2020). Using NaBH_4_ as the hydrogen source, the 3D reduced graphene oxide (rGO) aerogel with immobilized ultra-small Ag nanoparticles (1–3 nm) functions as a highly efficient heterogeneous catalyst for the hydrogenation of nitroaromatic compounds (such as nitrobenzene to aniline), where the Ag NPs serve as the main active sites for hydride (H^−^) transfer and electron mediation to activate and reduce the nitro group (Shen et al., 2020). The Ag nanoparticles anchored on graphene quantum dots (Ag/GQDs) serve as a highly efficient nano-catalyst for the reduction of nitro compounds (including nitrobenzene to aniline) using NaBH_4_ as the hydrogen source. The small Ag NPs act as active catalytic sites to facilitate electron transfer and hydride delivery, accelerating the nitro group reduction (Torabi et al., 2025).

#### 1.4.5 Rhodium based catalyst

**SCHEME 11:**
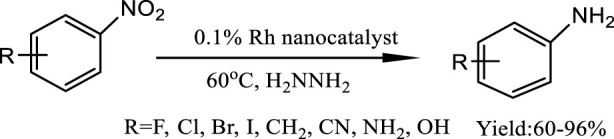
Reduction of nitrobenzene into aniline catalyzed by Rh catalyst (Luo et al., 2012).

Using formic acid or ammonium formate as a safe and environmentally friendly hydrogen source; the rhodium-terpyridine complex acts as a very effective homogeneous catalyst to create active rhodium-hydride intermediates. Under mild reaction conditions, Rh preferentially catalyzes the transfer hydrogenation of the nitro group in nitrobenzene, allowing for a clean stepwise reduction to aniline in water (Liu et al., 2021a; Tan et al., 2021). The main active site for the selective electrochemical hydrogenation of nitrobenzene in the work is rhodium (Rh) in atomically distributed Cu-coordinated Rh metallene arrays, which effectively activates and transfers electrons/protons to the nitro group under applied voltage (Mao et al., 2023; Sheng et al., 2023). Using hydrazine as the reducing agent and supported rhodium nanoparticles as the catalyst presents a highly effective and selective process for converting nitroarenes to amines. The study shows that this catalytic system is useful for synthesizing amines utilized in fine chemicals and pharmaceuticals because it produces great yields and selectivity under mild conditions (Chugh et al., 2022; Liu Z. et al., 2022; Luo et al., 2012) (Scheme 11).

Rh can activate and transfer hydrogen from hydrazine to the nitro group, allowing nitroarenes to be reduced to their corresponding amines selectively and efficiently under mild conditions. High catalytic activity and selectivity are caused by the maximum surface area and unique electrical properties of Rh nanoparticles, which enhance the efficiency and specificity of the reaction (Chen et al., 2022; Guha et al., 2014; Ruan et al., 2025).

#### 1.4.6 Ruthenium based catalysts

The RuNi single-atom alloy catalyst, which is composed of isolated Ru atoms anchored on Ni nanoparticles, uses Ru–Ni interfacial sites as its main active centers. Adjacent Ni sites effectively dissociate H_2_, allowing for quick and selective hydrogenation whereas negatively charged Ru^δ–^at these interfaces preferentially adsorbs nitrobenzene and encourages N–O bond breaking in the nitro group (Liao et al., 2023; Liu W. et al., 2022). Due to the significant differences in the adsorption strengths of the two chemical moieties on metal catalyst surfaces, the dual hydrogenation of nitro and aromatic groups poses special challenges. In a study of the hydrogenation of nitrobenzene as a model reaction, ruthenium supported on carbon nanotubes (Ru/CNT) catalysts provided the ideal equilibrium, allowing hydrogenation of both the aromatic ring and the nitro group. Methyl-labeled substrates made it possible to follow certain substrates and learn more about the relative rates at which nitrobenzene and its intermediates hydrogenate. Together with information on the bond between nitrobenzene and aniline on the Ru/CNT catalyst, an improved mechanistic model for the hydrogenation of nitrobenzene is presented (Liu Z. et al., 2022; Qusti et al., 2014; Taheri-Ledari et al., 2020).

Selective reduction of the nitro group in nitrobenzene and efficient H_2_ activation (from external H_2_ or *in-situ via* isopropanol dehydrogenation) are made possible by the bimetallic Ru-Ni nanoparticles on mesoporous carbon black. This leads to tandem coupling of hydrogenation and dehydrogenation in an aqueous medium at 240 °C (Li S. et al., 2025).

#### 1.4.7 Iron-based catalysts

**SCHEME 12:**
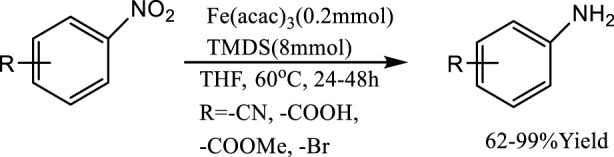
Selective reduction of nitroarenes catalyzed by Fe based catalyst.

Iron, nickel, and cobalt-containing magnetic nanoparticles are typically employed in the production of medical and data storage devices (Bahadur et al., 2025). By reducing the energy barriers for important intermediates (nitrosobenzene and phenylhydroxylamine) the Fe–N_4_ site aids the dissociative adsorption of H_2_ and encourages the stepwise reduction of the nitro group, allowing for effective conversion to aniline (Liu et al., 2021b; Tian et al., 2021). Fe(acac)_3_–catalyzed reduction of aromatic nitro compounds to anilines with TMDS in THF at 60 °C. High yields (62–99%) are obtained by tolerating sensitive groups such as -CN, -COOH, -COOM_e_, and -Br (Scheme 12). Ultra-small FeS_2_ nanoparticles serve as an efficient heterogeneous catalyst for the chemoselective transfer hydrogenation of nitroarenes using hydrazine hydrate as a safe hydrogen donor. Under mild conditions they achieve 99.9% selectivity to aniline derivatives by selectively converting the nitro group (-NO_2_) to the amino group (-NH_2_) *via* a neat stepwise pathway (nitroso → hydroxylamine → aniline). Without the need for gaseous H_2_, the catalyst’s ultra-small size, large surface area and pyrite structure guarantee superb activity, outstanding recyclability and wide functional group tolerance (Southouse et al., 2021). These catalysts are also used in typical processes like reduction and oxidation since they are simple to remove from the substrate (Deng et al., 2011; Park et al., 2007).

Iron oxide (Fe_3_O_4_) nanoparticles are inexpensive, reusable and commercially available. At 150 °C, the catalysts were used to effectively reduce nitro compounds in 2 min while employing hydrazine hydrate (N_2_H_4_.H_2_O) as a hydrogen source (Figure 3).

**FIGURE 1:**
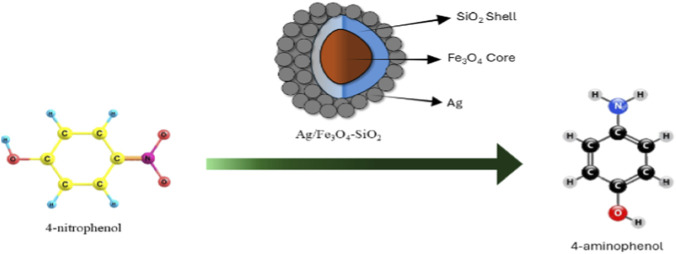
Schematic representation of the reduction of the 4-nitrophenol in the presence of Ag/Fe_3_O_4_-SiO_2_.

**FIGURE 2:**
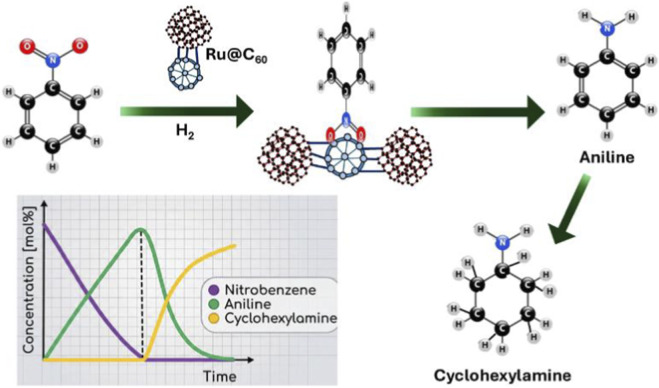
Hydrogenation of nitrobenzene catalyzed by Ru catalyst (Leng et al., 2016).

**FIGURE 3:**
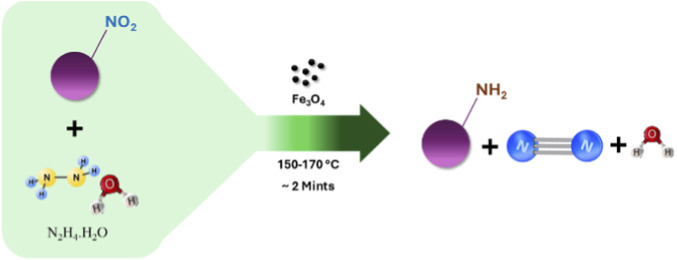
Iron oxide catalyzed efficiency for conversion of nitro compounds.

**FIGURE 4:**
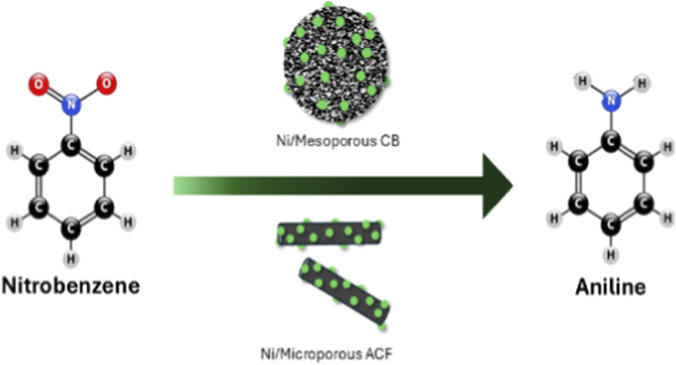
Conversion of nitrobenzene by Ni based catalyst.

Without seeing a significant drop in activity, the heterogeneous catalyst was reused multiple times. Additionally, because of their stability, the catalysts can be kept for a few weeks. It can then be used again to other reduction reactions (Cantillo et al., 2013; Huang et al., 2020; Rubab et al., 2024; Southouse et al., 2021).

#### 1.4.8 Nickel-based catalysts

Nickel (Ni) nanoparticles supported on carbon materials that have been acid-treated serve as the primary active phase in the gas-phase hydrogenation of nitrobenzene to aniline. They provide metallic Ni sites that dissociate molecular H_2_ into reactive atomic hydrogen and strongly adsorb the nitrobenzene molecule *via* its nitro group for effective activation (Nieto-Márquez et al., 2009; RAJ et al., 2012; Turáková et al., 2015). The nickel nanoparticles stabilized by filamentous carbon serve as the primary active catalytic sites for the hydrogenation of nitrobenzene to aniline. The metallic Ni (0) surface effectively separates molecular H_2_ into reactive atomic hydrogen and strongly adsorbs the nitro group of nitrobenzene, allowing chemo selective reduction under mild liquid-phase conditions (e.g., 100 °C, 2 MPa H_2_) (Mahata et al., 2008). Cost has always been a major factor in the development of new catalysts for a variety of processes. Due to the widespread use of noble metal catalysts, which has been constrained by their high cost, Ni-NPS has proven to be a strong alternative in terms of cost effectiveness, stability, strength and catalytic activity (Du et al., 2004; Meng et al., 2011). Through strong metal-support interactions (SMSI) with titanium oxide, nickel (Ni) in NiTiO_x_ catalysts creates Ni−O_v_−Ti sites (O_v_ = oxygen vacancies) which are essential for the selective reduction of nitroarenes to anilines. Hydrogen spillover to TiO_x_ for nitro group reduction is made possible by the effective dissociation of H_2_ by electron-rich Ni (Ni^δ−^). By giving nitro group adsorption priority and preventing adverse processes like dehalogenation, these sites guarantee high chemo selectivity (>90% yield for diverse nitroarenes). NiTiO_x_-500 which is calcined at 500 °C and balances Ni particle size (∼5 nm) and oxygen vacancies performs well. The catalyst has good activity and selectivity for aniline synthesis and is safer than Raney nickel. It is also stable and reusable with low loss over five cycles (Klausfelder and Kempe, 2023; Zhang C. et al., 2025). Nitrobenzene hydrogenation to aniline (C_6_H_5_NO_2_ + 3H_2_ → C_6_H_5_NH_2_ + 2H_2_O) is catalyzed by Ni, with performance differing depending on the support. The homogeneous ∼10 nm Ni particles and mesopores that enhance diffusion allow Ni on mesoporous carbon black (CB) to achieve ∼100% conversion and selectivity at 120 °C in 1 h. Because micropores restrict access, Ni on AC fiber (ACF) is slower (2 h). Due to fewer mesopores and poorer metal-support interactions, Ni on γ-Al_2_O_3_ at 150 °C, takes 2 hours to complete the reaction. The mesopores in CB increase Ni stability and decrease activation energy (42.6 kJ/mol vs. to 66.0 kJ/mol for Al_2_O_3_). Ni’s activity, selectivity and recyclability are maximized by mesoporous CB (Jiang et al., 2023; Loos et al., 2016; Qu et al., 2017).

Under mild conditions (room temperature, 1 atm H_2_), the nickel (Ni) in the three-dimensional network Pd-Ni/γ-Al_2_O_3_ catalyst functions as a crucial promoter and co-active metal, forming a Pd-Ni alloy or intimate bimetallic interface that alters the electronic properties of Pd, lowers the H_2_ dissociation barrier and increases the overall hydrogenation activity of nitrobenzene to aniline (Jiang et al., 2021; McCullagh et al., 2024). Using NaBH_4_, the two-dimensional Ni@Cu-MOF bimetallic nanosheets function as a non-noble metal catalyst effectively chemo selectively hydrogenates nitroarenes to corresponding anilines under mild conditions, achieving over 100% conversion and over 99% selectivity for a variety of substrates. While the ultrathin 2D structure optimizes atomic dispersion of active sites for improved catalytic efficiency, the Ni-Cu synergy prevents side reactions like dehalogenation by balancing reactivity (from Ni) and selectivity (from Cu) (Zhang et al., 2026). The nickel catalyst is the main active species in the hydrogenation of nitrobenzene to aniline (which can be Ni nanoparticles, Raney Ni, heterogeneous Ni-supported systems or homogeneous Ni complexes). It provides inexpensive, readily available metal sites that effectively activate molecular H_2_ (or transfer hydrogen donors) and coordinate the nitro group for chemo selective reduction under mild to moderate conditions (Nagy et al., 2025; Paul et al., 2025).

#### 1.4.9 Copper-based catalysts

**SCHEME 13:**
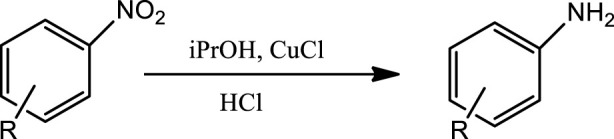
Reduction of nitrobenzene into anilines by using iPrOH, CuCl and HCl.

The copper catalyst is the active heterogeneous phase for the vapor-phase hydrogenation of nitrobenzene to aniline (usually industrial supported Cu such as Cu on Al_2_O_3_ or similar) which provides metallic Cu sites that dissociate molecular H_2_ into adsorbed hydrogen atoms and enable the adsorption of nitrobenzene *via* its nitro group (Larsen et al., 2000; Petrov et al., 1986; Petrov et al., 1990). By providing metallic Cu sites that strongly adsorb nitrobenzene and dissociate H_2_ into reactive hydrogen species, the copper catalyst (supported Cu e.g., Cu/SiO_2_ or industrial Cu-based systems) plays a crucial role in the proposed new mechanism. This allows direct stepwise hydrogenation of the nitro group to the amino group in a manner different from the conventional Haber pathway (Gelder et al., 2005; Moran et al., 2020b; Yánez et al., 2006). Certain reduction kinetics is more suited for Cu-based catalysts since they may reduce the dynamic obstacle in the response. When they are expanding, they are quite sensitive to nitrogen-containing groups. They do not affect the ester, halogen or carbonyl groups but they do transform the nitro and nitrile groups into important amines (Stergiou et al., 2022; Toshima and Wang, 1994). For example, 3-nitro-4-methoxy-acetylaniline (NMA) was converted to 3-amino-4-methoxy-acetylaniline (AMA) when copper nanoparticles were present (Toshima and Wang, 1994). Isopropanol (iPrOH) is used as a green hydrogen source in aqueous HCl/iPrOH at room temperature to convert nitro benzenes to anilines *via* a visible light-photoinduced and Cu-catalyzed process. Nitrosobenzene and N-phenylhydroxylamine serve as intermediates in the process. Initial reductions are driven by photoexcitation and hydrogen abstraction in the presence of visible light and iPrOH. To facilitate effective proton-coupled electron transfers in the direct pathway (nitro → nitroso benzene → phenylhydroxylamine → aniline) with moderate binding to prevent poisoning or high barriers, copper (Cu) particularly on the Cu(111) surface is crucial in balancing the adsorption energies of nitrobenzene reduction intermediates *via* DFT (Density Functional Theory) (Martina et al., 2023; Wong et al., 2022) (Scheme 13). The copper (Cu) in the bimetallic Cu-Ni nanoparticles supported on AC has a synergistic role in the catalytic transfer hydrogenation of nitroarenes including nitrobenzene to anilines. To improve the activation of the hydrogen donor (such as isopropanol or comparable alcohols) Cu helps alter the electronic characteristics of Ni by decreasing the size of Ni particles, enhancing dispersion and raising overall catalytic reducibility (Guan et al., 2021; Kimi et al., 2018). Copper nanoparticles (Cu NPs) supported on doped graphitic carbon are the primary active sites for the reduction of nitroaromatics, particularly nitrobenzene to amines. To create active hydrogen species that drive the chemo-selective hydrogenation of the nitro group, the metallic Cu (0) nanoparticles efficiently dissolve NaBH_4_ (or other reductants) (Mirhosseyni et al., 2021; Nandi et al., 2017). To prevent adverse reactions such as arene chlorination, N-phenylhydroxylamine is converted to aniline by Cu(I) salt. The Cu(I)-Cu (II) catalytic cycle is supported and the reduction is accelerated by aqueous HCl. C_6_H_5_NO_2_ + 6H → C_6_H_5_NH_2_ + 2H_2_O is the complete reaction. Aqueous HCl, Cu(I) salt (such as CuCl) and iPrOH are important reagents. The approach is effective and selective (Bal et al., 2025).

Cu(I) is an essential catalyst that according to the study, permits effective and selective reduction while preserving a regenerative catalytic cycle (Bal et al., 2025).

#### 1.4.10 Cobalt based catalyst

**SCHEME 14:**
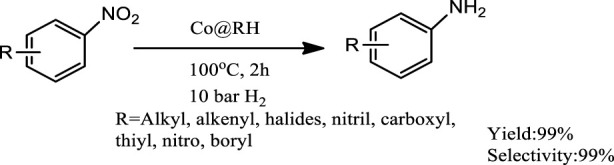
Conversion of nitrobenzene into aniline by Co@RH (Unglaube et al., 2022).

The reduction of nitrobenzene to aniline is effectively catalyzed by the nickel cobalt oxide (NiCo_2_O_4_) nanowire-reduced graphite oxide (rGO) composite where the mixed Ni–Co spinel oxide provides redox-active metal sites (Ni^2+^/Ni^3+^ and Co^2+^/Co^3+^) that enable H_2_ dissociation and activation under mild conditions (Kondeboina et al., 2018; Wang et al., 2014). The characteristics of the N-doped carbon-supported cobalt single-atom catalyst (Co–N4/C)-catalyzed reduction of nitrobenzene to aniline. Computed findings show that the reduction of nitrobenzene to aniline is significantly influenced by the presence of water molecules (Qi et al., 2024; Wang et al., 2021; Zhou et al., 2017). The high activity of the cobalt catalyst is caused by the combined action of Co NPs and the biochar support, which provides a high surface area (up to 400 m^2^/g) and stabilizes active sites. With a turnover frequency (TOF) of about 120 h^-1^, the catalyst performed better under comparable conditions than both noble metal catalysts (such Pd/C) and commercial Co-based catalysts. After five cycles, the catalyst showed good stability with low cobalt leaching (<1 ppm) and retained >95% conversion and selectivity (Gutiérrez-Tarriño et al., 2021; Unglaube et al., 2022) (Scheme 14). By enhancing Pd nanoparticle dispersion and generating electron-rich Pd sites through amine coordination which fortifies nitro group adsorption and speeds up H_2_ dissociation, the amine-functionalized cobalt-ferrite-supported palladium catalyst (Pd/CoFe_2_O_4_-NH_2_) greatly improves the hydrogenation of nitrobenzene to aniline (Hajdu et al., 2023; Morisse et al., 2021). The electron-rich corrole ligand helps the nitro group of nitrobenzene coordinate to the Co(III) center, stabilizing the substrate-catalyst interaction (Lu et al., 2022). A transitory Co-H species is formed when the cobalt core activates molecular H_2_, most likely through heterolytic cleavage. This procedure is improved by the DMSO ligand, which modifies the cobalt’s electrical environment (Petrelli et al., 2023; Timelthaler et al., 2021).

The single-atom cobalt (Co SAC) catalyst, which has isolated Co atoms coordinated in N-doped carbon (often Co-N_4_ sites), is the extremely active and chemo selective center for the hydrogenation of nitroarenes to anilines. In mild conditions (e.g., 100 °C, 20 bar H_2_), this enables significant adsorption of the nitro group and efficient activation of H_2_ (Amirjan et al., 2024; Liu et al., 2023; Qi et al., 2024).

#### 1.4.11 Mo based catalysts

**SCHEME 15:**
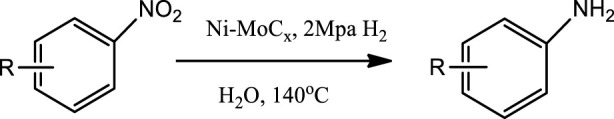
Conversion of nitrobenzene into aniline by Ni-MoC (Zhang et al., 2023b).

The well-defined homogeneous catalyst is the Mo_3_S_4_ cuboidal molybdenum sulfide cluster (more precisely the diamino complex [Mo_3_S_4_Cl_3_ (dmen)_3_]^+^, where dmen = N,N′-dimethyl ethylenediamine) with the molybdenum centers acting as the primary active sites for activating hydrogen (H_2_) and enabling the stepwise reduction of nitro compounds to aniline intermediates (Pedrajas et al., 2017; Wang et al., 2016). The main active phase of the Co-Mo_2_C/AC catalyst is molybdenum carbide (Mo_2_C) which has Pt-like electrical characteristics and plenty of surface sites for H_2_ dissociation and activation. This allows the nitro group in nitrobenzene to be efficiently chemo selectively hydrogenated to aniline (Zhao et al., 2014). The primary active phase of the Mo_2_C/NC catalyst is molybdenum carbide (Mo_2_C) where the Mo sites show Pt-like activity for effective H_2_ dissociation and activation, allowing nitrobenzene to be chemo selectively hydrogenated to aniline under mild conditions (Liu S. et al., 2021; Luo et al., 2021; Ma et al., 2021). High activity for nitrobenzene hydrogenation was demonstrated by the Ni-MoC_x_ catalyst which was made by carbonizing a NiMo-metal-organic framework (MOF) precursor. Under optimum conditions (1 MPa H_2_, 100 °C and ethanol solvent) the catalyst converted nitrobenzene to aniline with >99% selectivity in 4 h. The synergistic interaction between Ni nanoparticles and MoC_x_ (molybdenum carbide) species which promotes hydrogen activation and nitro group reduction, is responsible for high activity (Phasayavan et al., 2024; Zhang Y. et al., 2023) (Scheme 15).

Using hydrazine hydrate as the hydrogen donor, molybdenum trioxide (MoO_3_) is a highly effective heterogeneous catalyst for the chemo-selective reduction of nitroarenes including nitrobenzene to arylamines (anilines) in mild conditions. MoO_3_ reacts with hydrazine hydrate to form an active *in-situ* complex (probably involving reduced Mo species or Mo-hydrazine intermediates) that promotes the production of active hydrogen species. This enables the nitro group (-NO_2_) to be gradually reduced to the amino group (-NH_2_) with high yields (>95%) and outstanding selectivity (Maurya et al., 2024; Phasayavan et al., 2024).

#### 1.4.12 Mn based catalysts

**SCHEME 16:**
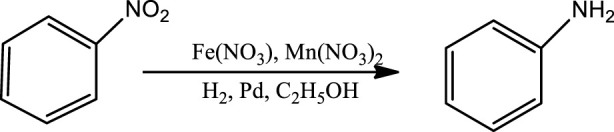
Conversion of nitrobenzene into Aniline by Mn catalysts (Hajdu et al., 2022).

Porous manganese oxide (OMS-2) is the primary active support in the hydrogenation of nitrobenzene to aniline. It contains redox-active Mn oxidation state typically include (Mn^+4/^Mn^3+^/Mn^2+^) that promote stepwise reduction *via* intermediates such as azobenzene/hydrazobenzene, azoxybenzene and nitrosobenzene, activate the nitro group and facilitate oxygen mobility (McManus et al., 2016; Yadav and Mewada, 2013). The manganese oxide supported on partially reduced graphene oxide (MnO_2_/prGO) catalyst allows the selective hydrogenation of nitrobenzene to aniline by activating molecular hydrogen (H_2_) on Mn active sites which spread over to the graphene support for enhanced electron transfer and reactant adsorption (Rana et al., 2021). Through coordinative binding at the Mn core the air-stable manganese(I) pincer complex catalyst activates molecular hydrogen (H_2_) enabling the selective hydrogenation of nitrobenzene to aniline with high yields (59%–99%) (Zubar et al., 2021). By activating H_2_ on Pd sites under mild conditions (40 °C, 10 bar), the Pd/MnFe_2_O_4_ catalyst, which has palladium on manganese-ferrite, selectively hydrogenates nitrobenzene to aniline (>99% yield). Pd dispersion and electron transport are improved by the MnFe_2_O_4_ support which facilitates effective nitro group reduction while preserving excellent selectivity. For up to ten cycles, its magnetic separability guarantees simple recovery and reusability with little activity loss (Hajdu et al., 2022) (Scheme 16). By activating silanes (such as PhSiH_3_) to produce reactive manganese-hydride species the Mn (II)-NNO pincer complex functions as a well-defined homogeneous catalyst that permits the progressive reduction of the nitro group in nitrobenzene to aniline *via* chemo selective hydrosilylation (Behera et al., 2022). Under mild conditions (100 °C–150 °C) manganese, iron and cobalt pincer complexes (e.g., Mn-PNP) catalyze selective nitroarene reduction to primary amines with up to 99% yield, either by activating H_2_ or by employing transfer hydrogenation. These catalysts tolerate sensitive functional groups like halides and ketones by enabling stepwise nitro group reduction through metal-ligand collaboration. Thus, with silanes such as PhSiH_3_ on activation form these reactive manganese-hydride species. The Mn(II)-NNO pincer complex is then used as a homogeneous catalyst without needing additional additives and the nitro group in nitrobenzene is reduced step-by-step, finally to get aniline by chemo selective hydrosilylation (Wu and Darcel, 2023). For the chemo selective hydrogenation of nitroarenes the MnO_X_ nanocatalyst based on Al_2_O_3_ functions as a highly active heterogeneous catalyst. It efficiently activates molecular hydrogen (H_2_) on its surface reducing the nitro group (-NO_2_) to the amine (-NH_2_) (Rana et al., 2025).

The (NHC)Mn(I) complex, or more specifically, a phosphine-free bis-NHC-Mn(I) complex, functions as an effective well-defined homogeneous catalyst for the selective transfer hydrogenation of nitrobenzene to aniline when ammonia borane (AB) is used as the hydrogen donor (Kumari et al., 2024). Because of highly labile Mn–O coordination bonds which easily permit the creation of catalytically active sites, the MnL_4_ manganese-based coordination complex serves as a very effective photocatalyst for the reduction of nitrobenzene to aniline under visible light. In comparison to Mn_4_L_4_ which has stronger Mn–O bonds that prevent the development of active sites and lead to decreased activity, the flexible Mn_4_L_4_ microenvironment facilitates the creation of open manganese sites for substrate activation and electron/hydride transfer (Xu et al., 2026).

### 1.5 Conclusion

Using heterogeneous metal-based nanocatalysts to catalytically convert nitroarenes to amino arenes is a breakthrough in environmental remediation and synthetic organic chemistry. The synthesis of noble (Pd, Pt, Au, Rh and Ru) and non-noble (Fe, Co, Ni, Cu, Mo and Mn) catalysts that are efficient, selective and long-lasting metal nanoparticles supported by a variety of materials like metal oxides, metal-organic frameworks (MOFs) and carbonaceous supports, has advanced remarkably, as this review demonstrates. Single-atom catalysts, plasmonic nanomaterials and bimetallic alloys are examples of innovations that have improved catalytic performance and made it possible to reduce nitro under mild reaction conditions using hydrogen, hydrazine, NaBH_4_ or photocatalytic and electrochemical techniques. High conversion rates (up to 100%), superior selectivity (e.g., 82%–99%) and quick reaction times (as low as 1.5–8 min) for important transformations like nitrobenzene to aniline are noteworthy accomplishments. The drive toward sustainability is further supported by environmentally friendly methods like solvent-free processes, magnetically recoverable catalysts and green hydrogen sources like glycerol. Functionalized carbon nanotubes and nitrogen/oxygen-doped activated carbon are two examples of carbon-based catalysts that have shown promise as metal-free substitutes that are both economical and environmentally beneficial. Scalability, cost-effectiveness and long-term catalyst stability are still major obstacles despite these developments. To completely exploit the industrial and environmental potential of these catalytic processes, future research should concentrate on establishing scalable, economical and long-lasting systems, expanding mechanistic knowledge and optimizing nanocatalyst design. This paper offers a thorough road map for developing nitroarene reduction, opening the door to more environmentally friendly and effective chemical synthesis. The study of heterogeneous metal-based nanocatalysts has led to significant advancements in the catalytic reduction of nitroarenes to amino arenes. Non-noble metal systems particularly those based on Ni, Cu, Co, and Fe offer a very promising future because they are much less expensive, highly active, completely heterogeneous and readily recoverable (through magnetic separation or filtration). They also provide excellent conversion (up to 100%), selectivity (>99%) and reusability under mild, sustainable conditions. To fully realize their industrial and environmental potential for more environmentally friendly amino arenes synthesis, future efforts should focus on designing photocatalysts that are affordable, highly active, scalable and ultra-stable. The remarkable improvements in nanocatalyst engineering are highlighted in this study, especially the development of non-noble metal catalysts that exhibit exceptional activity, selectivity and reusability under moderate conditions. The knowledge covered here provides a useful road map for developing sustainable catalytic systems for use in industry and ecological remediation.

## References

[B1] Aday B. Yıldız Y. Ulus R. Eris S. Sen F. Kaya M. (2016). One-pot, efficient and green synthesis of acridinedione derivatives using highly monodisperse platinum nanoparticles supported with reduced graphene oxide. New J. Chem. 40 (1), 748–754. 10.1039/c5nj02098k

[B2] Akbarizad B. Taghavi M. Najafi S. (2024). Selective reduction of nitroaromatic compounds to their corresponding amine compounds using NaBH_4_/Ni (PPh_3_)_4_ . J. Synthetic Chem. 3 (2), 110–120. 10.22034/jsc.2024.463857.1078

[B3] Amirjan M. Nemati F. Elahimehr Z. Rangraz Y. (2024). Copper oxides supported sulfur-doped porous carbon material as a remarkable catalyst for reduction of aromatic nitro compounds. Sci. Rep. 14 (1), 5491. 10.1038/s41598-024-55216-0 38448558 PMC10918164

[B4] Anusuya N. Pragathiswaran C. Mary J. V. (2021). A potential catalyst-TiO_2_/ZnO based chitosan gel beads for the reduction of nitro-aromatic compounds aggregated sodium borohydride and their antimicrobial activity. J. Mol. Struct. 1236, 130197. 10.1016/j.molstruc.2021.130197

[B5] Aprile G. Szakter K. Byrholtz Andersen M. Wu H. Sin G. Skovby T. (2024). Continuous spherical crystallization of escitalopram oxalate without additives. Org. Process Res. Dev. 28 (2), 532–542. 10.1021/acs.oprd.3c00345

[B6] Bahadur V. Dehade A. S. Das D. Kamath P. Pal S. Manjunath B. (2025). Iron–water mediated chemoselective reduction of nitroarenes in a ball mill: a highly efficient and sustainable approach. RSC Mechanochem. 2 (6), 802–808. 10.1039/d5mr00055f

[B7] Bal M. Van Hoey W. Cleirbaut R. Lemière F. Van Doorslaer S. Cool P. (2025). Visible light-photoinduced and Cu-catalyzed reduction of nitrobenzenes into anilines. ACS Catal. 15 (6), 4726–4738. 10.1021/acscatal.4c07841

[B8] Behera R. R. Panda S. Ghosh R. Kumar A. A. Bagh B. (2022). Manganese-catalyzed chemoselective hydrosilylation of nitroarenes: sustainable route to aromatic amines. Org. Lett. 24 (50), 9179–9183. 10.1021/acs.orglett.2c03576 36413437

[B9] Boyall S. L. Berman P. Griffiths A. Massey A. Dixon T. Shaw T. (2024). Palladium nanoparticle deposition on spherical carbon supports for heterogeneous catalysis in continuous flow. Catal. Sci. Technol. 14 (9), 2563–2573. 10.1039/d3cy01718d

[B10] Campbell J. McCullagh A. McGrath L. How C. MacLaren D. Loenders M. (2024). The application of alumina supported Pd catalysts for high selectivity aniline synthesis catalysis at elevated temperatures: site-selective chemistry. Appl. Catal. A General 670, 119541. 10.1016/j.apcata.2023.119541

[B11] Campos C. H. Shanmugaraj K. Bustamante T. M. Leal-Villarroel E. Vinoth V. Aepuru R. (2022). Catalytic production of anilines by nitro-compounds hydrogenation over highly recyclable platinum nanoparticles supported on halloysite nanotubes. Catal. Today 394, 51a–523. 10.1016/j.cattod.2021.06.027

[B12] Cantillo D. Moghaddam M. M. Kappe C. O. (2013). Hydrazine-mediated reduction of nitro and azide functionalities catalyzed by highly active and reusable magnetic iron oxide nanocrystals. J. Org. Chem. 78 (9), 4530–4542. 10.1021/jo400556g 23560824

[B13] Capperucci A. Clemente M. Cenni A. Tanini D. (2023). Transition metal‐free Selenium‐mediated Aryl amines *via* reduction of nitroarenes. ChemSusChem 16 (15), e202300086. 10.1002/cssc.202300086 36971384

[B14] Cárdenas-Lizana F. Berguerand C. Yuranov I. Kiwi-Minsker L. (2013). Chemoselective hydrogenation of nitroarenes: boosting nanoparticle efficiency by confinement within highly porous polymeric framework. J. Catal. 301, 103–111. 10.1016/j.jcat.2013.01.021

[B15] Chen W. Li H. Jin Y. Wu C. Yuan Z. Ma P. (2022). An intriguing tetranuclear Rh-based polyoxometalate for the reduction of nitroarene and oxidation of aniline. Chem. Commun. 58 (71), 9902–9905. 10.1039/d2cc03076d 35975716

[B16] Choi Y. Bae H. S. Seo E. Jang S. Park K. H. Kim B. S. (2011). Hybrid gold nanoparticle-reduced graphene oxide nanosheets as active catalysts for highly efficient reduction of nitroarenes. J. Mater. Chem. 21 (39), 15431–15436. 10.1039/c1jm12477c

[B17] Chugh V. Chatterjee B. Chang W. C. Cramer H. H. Hindemith C. Randel H. (2022). An adaptive rhodium catalyst to control the hydrogenation network of nitroarenes. Angew. Chem. Int. Ed. 61 (36), e202205515. 10.1002/anie.202205515 PMC9544374 35759682

[B18] Corma A. Garcia H. (2008). Supported gold nanoparticles as catalysts for organic reactions. Chem. Soc. Rev. 37 (9), 2096–2126. 10.1039/b707314n 18762848

[B19] Corma A. Serna P. (2006). Chemoselective hydrogenation of nitro compounds with supported gold catalysts. Science 313 (5785), 332–334. 10.1126/science.1128383 16857934

[B20] d'Andrea L. Kristensen J. L. (2023). One-pot reduction of nitrostyrenes to phenethylamines using sodium borohydride and copper (II) chloride. 10.3762/bjoc.21.4 PMC11729678 39811686

[B21] Daems N. Wouters J. Van Goethem C. Baert K. Poleunis C. Delcorte A. (2018). Selective reduction of nitrobenzene to aniline over electrocatalysts based on nitrogen-doped carbons containing non-noble metals. Appl. Catal. B Environ. 226, 509–522. 10.1016/j.apcatb.2017.12.079

[B22] Davis S. E. Ide M. S. Davis R. J. (2013). Selective oxidation of alcohols and aldehydes over supported metal nanoparticles. Green Chem. 15 (1), 17–45. 10.1039/c2gc36441g

[B23] Deng J. Mo L. P. Zhao F. Y. Hou L. L. Yang L. Zhang Z. H. (2011). Sulfonic acid supported on hydroxyapatite-encapsulated-γ-Fe_2_O_3_ nanocrystallites as a magnetically separable catalyst for one-pot reductive amination of carbonyl compounds. Green Chem. 13 (9), 2576–2584. 10.1039/c1gc15470b

[B24] Doherty S. Knight J. G. Alharbi H. Y. Paterson R. Wills C. Dixon C. (2025). Gold nanoparticle‐catalyzed solvent switchable selective partial reduction of nitrobenzene to N‐Phenylhydroxylamine and Azoxybenzene. ChemCatChem 17 (5), e202401702. 10.1002/cctc.202401702

[B25] Du Y. Chen H. Chen R. Xu N. (2004). Synthesis of p-aminophenol from p-nitrophenol over nano-sized nickel catalysts. Appl. Catal. A General 277 (1-2), 259–264. 10.1016/j.apcata.2004.09.018

[B26] Fang B. Qi Z. Liu F. Zhang C. Li C. Ni J. (2022). Activity enhancement of ceria-supported Co-Mo bimetallic catalysts by tuning reducibility and metal enrichment. J. Catal. 406, 231–240. 10.1016/j.jcat.2022.01.015

[B27] Feng F. Zhen Y. Chen Y. Luo J. Yao C. Pan L. (2024). Size control of Pt nanoparticle catalysts for high-selectivity hydrogenation of nitrobenzene to p-aminophenol. Mol. Catal. 556, 113922. 10.1016/j.mcat.2024.113922

[B28] Formenti D. Ferretti F. Scharnagl F. K. Beller M. (2018). Reduction of nitro compounds using 3d-non-noble metal catalysts. Chem. Rev. 119 (4), 2611–2680. 10.1021/acs.chemrev.8b00547 30516963

[B29] Fu X. Qi Z. Ren W. Xu M. Yang Y. (2023). Photocatalytic reduction of nitrobenzene to aniline over CdS nanorods: the impacts of reaction conditions and the hydrogenation mechanism. Catal. Sci. Technol. 13 (24), 7022–7035. 10.1039/d3cy01252b

[B30] Fujita S.-i. Watanabe H. Katagiri A. Yoshida H. Arai M. (2014). Nitrogen and oxygen-doped metal-free carbon catalysts for chemoselective transfer hydrogenation of nitrobenzene, styrene, and 3-nitrostyrene with hydrazine. J. Mol. Catal. A Chem. 393, 257–262. 10.1016/j.molcata.2014.06.021

[B31] Gawande M. B. Rathi A. K. Branco P. S. Nogueira I. D. Velhinho A. Shrikhande J. J. (2012). Regio‐and chemoselective reduction of nitroarenes and carbonyl compounds over recyclable magnetic ferrit nickel nanoparticles (Fe_3_O_4_ ni) by using glycerol as a hydrogen source. Chemistry-a Eur. J. 18 (40), 12628–12632. 10.1002/chem.201202380 22933355

[B32] Gelder E. A. Jackson S. D. Lok C. M. (2005). The hydrogenation of nitrobenzene to aniline: a new mechanism. Chem. Commun. (4), 522–524. 10.1039/b411603h 15654390

[B33] Genc H. (2015). Efficient reductions of various nitroarenes with scrap automobile catalyst and NaBH4. Catal. Commun. 67, 64–67. 10.1016/j.catcom.2015.04.008

[B34] Gogoi M. Gogoi P. Borah P. Sarma D. Deori K. (2024). Catalytic versatility of lead-free Cu2+-doped Cs2AgBiCl6 double perovskite in sustainable photocatalysis and quinazoline synthesis. ACS Appl. Opt. Mater. 2 (11), 2359–2370. 10.1021/acsaom.4c00377

[B35] Guan X. Zhu H. Driver T. G. (2021). Cu-catalyzed cross-coupling of nitroarenes with aryl boronic acids to construct diarylamines. ACS Catalysis 11 (20), 12417–12422. 10.1021/acscatal.1c03113 35433104 PMC9007557

[B36] Guha N. R. Bhattacherjee D. Das P. (2014). Solid supported rhodium (0) nanoparticles: an efficient catalyst for chemo-and regio-selective transfer hydrogenation of nitroarenes to anilines under microwave irradiation. Tetrahedron Lett. 55 (18), 2912–2916. 10.1016/j.tetlet.2014.03.047

[B37] Guo J. Liu H. Li Y. Li D. He D. (2023). Recent advances on catalysts for photocatalytic selective hydrogenation of nitrobenzene to aniline. Front. Chem. 11, 1162183. 10.3389/fchem.2023.1162183 36970401 PMC10036363

[B38] Gutiérrez-Tarriño S. Rojas-Buzo S. Lopes C. W. Agostini G. Calvino J. J. Corma A. (2021). Cobalt nanoclusters coated with N-doped carbon for chemoselective nitroarene hydrogenation and tandem reactions in water. Green Chem. 23 (12), 4490–4501. 10.1039/d1gc00706h

[B39] Hajdu V. Muránszky G. Nagy M. Kopcsik E. Kristály F. Fiser B. (2022). Development of high-efficiency, magnetically separable palladium-decorated manganese-ferrite catalyst for nitrobenzene hydrogenation. Int. J. Mol. Sci. 23 (12), 6535. 10.3390/ijms23126535 35742977 PMC9224514

[B40] Hajdu V. Prekob Á. Muránszky G. Kristály F. Daróczi L. Harasztosi L. (2023). Amine functionalization leads to enhanced performance for nickel-and cobalt-ferrite-supported palladium catalysts in nitrobenzene hydrogenation. Int. J. Mol. Sci. 24 (17), 13347. 10.3390/ijms241713347 37686152 PMC10487572

[B41] Han J. Li L. Guo R. (2010). Novel approach to controllable synthesis of gold nanoparticles supported on polyaniline nanofibers. Macromolecules 43 (24), 10636–10644. 10.1021/ma102251e

[B42] Hasan K. Shehadi I. A. Joseph R. G. Patole S. P. Elgamouz A. (2023). β-Cyclodextrin-functionalized Fe3O4-supported Pd-nanocatalyst for the reduction of nitroarenes in water at mild conditions. ACS Omega 8 (26), 23901–23912. 10.1021/acsomega.3c02332 37426276 PMC10324381

[B43] Hasan I. M. u. Xu N. Liu Y. Nawaz M. Z. Feng H. Qiao J. (2024). Noble and non-noble metal based catalysts for electrochemical nitrate reduction to ammonia: activity, selectivity and stability. Electrochem. Energy Rev. 7 (1), 36. 10.1007/s41918-024-00236-7

[B44] He T. Zhang C. Zhang L. Du A. (2019). Single Pt atom decorated graphitic carbon nitride as an efficient photocatalyst for the hydrogenation of nitrobenzene into aniline. Nano Res. 12 (8), 1817–1823. 10.1007/s12274-019-2439-z

[B45] Herrera J. Gamallo P. Campos C. H. Alonso G. (2025). The importance of solvent adsorption in liquid‐phase reaction kinetics: nitrobenzene hydrogenation in Pd (111) as a case Study. ChemPhysChem 26, 2500110. 10.1002/cphc.202500110 40302229

[B46] Hoseini Chopani S. M. Asadi S. Heravi M. M. (2020). Application of bimetallic and trimetallic nanoparticles supported on graphene as novel heterogeneous catalysts in the reduction of nitroarenes, homo-coupling, Suzuki-Miyaura and Sonogashira reactions. Curr. Org. Chem. 24 (19), 2216–2234. 10.2174/1385272824999200914111559

[B47] Huang Y. Lin H. Zhang Y. (2020). Synthesis of MIL-101 (Fe)/SiO_2_ composites with improved catalytic activity for reduction of nitroaromatic compounds. J. Solid State Chem. 283, 121150. 10.1016/j.jssc.2019.121150

[B48] Hung T.-Y. Peng W.-S. Tang J.-W. Tsai F.-Y. (2022). A reusable FeCl_3_∙ 6H_2_O/Cationic 2, 2′-Bipyridyl catalytic system for reduction of nitroarenes in water. Catalysts 12 (8), 924. 10.3390/catal12080924

[B49] Islam I. U. Zhang Y. Dong B. Iqbal A. Abbas S. Zai J. (2024). Highly selective electroreduction of nitrobenzene to aniline by co-doped 1T-MoS2. ACS Appl. Mater. Interfaces 16 (19), 25090–25100. 10.1021/acsami.4c01425 38709646

[B50] Ji Y. Pan J. Dauenhauer P. Gorte R. J. (2019). Probing direct carbon-carbon acylation of furans and long-chain acids over H-ZSM-5. Appl. Catal. A General 577, 107–112. 10.1016/j.apcata.2019.03.012

[B51] Jiang Y. Li Q. Li X. Wang X. Dong S. Li J. (2021). Three-dimensional network Pd-Ni/γ-Al_2_O_3_ catalysts for highly active catalytic hydrogenation of nitrobenzene to aniline under mild conditions. ACS Omega 6 (14), 9780–9790. 10.1021/acsomega.1c00441 33869958 PMC8047756

[B52] Jiang H. Yuan G. Cui Z. Zhao Z. Dong Z. Zhang J. (2023). Effects of support types and their porosity characteristics on the catalytic performance of Ni-based catalysts in nitrobenzene hydrogenation to aniline. Industrial Eng. Chem. Res. 62 (34), 13355–13367. 10.1021/acs.iecr.3c01327

[B53] Jin M. Liu Y. Zhang X. Wang J. Zhang S. Wang G. (2021). Selective electrocatalytic hydrogenation of nitrobenzene over copper-platinum alloying catalysts: experimental and theoretical studies. Appl. Catal. B Environ. 298, 120545. 10.1016/j.apcatb.2021.120545

[B54] Kantam M. L. Reddy R. S. Srinivas K. Chakravarti R. Sreedhar B. Figueras F. (2012). Platinum nanoparticles supported on zirconia mediated synthesis of N-acyl and N-(tert-butoxycarbonyl) amines from nitroarenes and azides. J. Mol. Catal. A Chem. 355, 96–101. 10.1016/j.molcata.2011.12.002

[B55] Khaleghi N. Forouzandeh-Malati M. Ganjali F. Rashvandi Z. Zarei-Shokat S. Taheri-Ledari R. (2023). Silver-assisted reduction of nitroarenes by an Ag-embedded curcumin/melamine-functionalized magnetic nanocatalyst. Sci. Rep. 13 (1), 5225. 10.1038/s41598-023-32560-1 36997564 PMC10063568

[B56] Khanra M. Mhate M. Swain S. P. (2025). Potassium tert‐butoxide mediated benzyl radical generation from halo compounds and multi‐component reactions with selenourea and carboxylic acids for the synthesis of selenoesters. ChemCatChem 17, e202500123. 10.1002/cctc.202500123

[B57] Kimi M. Jaidie M. M. H. Pang S. C. (2018). Bimetallic Cu-Ni nanoparticles supported on activated carbon for catalytic oxidation of benzyl alcohol. J. Physics Chemistry Solids 112, 50–53. 10.1016/j.jpcs.2017.09.008

[B58] Klausfelder B. Kempe R. (2023). A highly active nickel catalyst for the selective hydrogenation of functionalized nitroarenes. Z. für Anorg. Allg. Chem. 649 (14), e202300071. 10.1002/zaac.202300071

[B59] Koga H. Kitaoka T. (2011). One-step synthesis of gold nanocatalysts on a microstructured paper matrix for the reduction of 4-nitrophenol. Chem. Eng. J. 168 (1), 420–425. 10.1016/j.cej.2010.08.073

[B60] Kominami H. Tanimoto K. Tanaka A. (2025). Continuous production of aminobenzenes by hydrogenation of nitrobenzenes over a TiO_2_ photocatalyst in a spiral flow reactor. Appl. Catal. A General 701, 120325. 10.1016/j.apcata.2025.120325

[B61] Kondeboina M. Enumula S. S. Gurram V. R. B. Yadagiri J. Burri D. R. Kamaraju S. R. R. (2018). Mesoporous silica supported cobalt catalysts for gas phase hydrogenation of nitrobenzene: role of pore structure on stable catalytic performance. New J. Chem. 42 (19), 15714–15725. 10.1039/c8nj03211d

[B62] Kumar S. Dholakiya B. Z. Jangir R. (2023). Covalent organic framework impregnated with silver and copper nanoparticles: an advanced approach for catalytic degradation of organic pollutants in wastewater. ACS Appl. Mater. Interfaces 16 (1), 1553–1563. 10.1021/acsami.3c15766 38159077

[B63] Kumar A. Savanur M. R. S. Singh S. K. Singh J. Bhattacharya S. Asnani K. (2025). Recent advances in silver nanoparticles‐catalyzed reactions. ChemistrySelect 10 (5), e202403655. 10.1002/slct.202403655

[B64] Kumari S. Roy S. Ranjan Saha K. Kundu S. (2024). (NHC) Mn (I) complex catalyzed selective transfer hydrogenation of epoxides, azoarenes and nitroarenes utilizing Ammonia borane. ChemCatChem 16 (21), e202400901. 10.1002/cctc.202400901

[B65] Lara P. Philippot K. (2014). The hydrogenation of nitroarenes mediated by platinum nanoparticles: an overview. Catal. Sci. Technol. 4 (8), 2445–2465. 10.1039/c4cy00111g

[B66] Lara P. Suárez A. Collière V. Philippot K. Chaudret B. (2014). Platinum n‐heterocyclic carbene nanoparticles as new and effective catalysts for the selective hydrogenation of nitroaromatics. ChemCatChem 6 (1), 87–90. 10.1002/cctc.201300821

[B67] Larsen J. W. Freund M. Kim K. Y. Sidovar M. Stuart J. L. (2000). Mechanism of the carbon catalyzed reduction of nitrobenzene by hydrazine. Carbon 38 (5), 655–661. 10.1016/s0008-6223(99)00155-4

[B68] Leng F. Gerber I. C. Lecante P. Moldovan S. Girleanu M. Axet M. R. (2016). Controlled and chemoselective hydrogenation of nitrobenzene over ru@ C60 catalysts. ACS Catal. 6 (9), 6018–6024. 10.1021/acscatal.6b01429

[B69] Li J. Shi X. Y. Bi Y. Y. Wei J. F. Chen Z. G. (2011). Pd nanoparticles in ionic liquid brush: a highly active and reusable heterogeneous catalytic assembly for solvent-free or on-water hydrogenation of nitroarene under mild conditions. ACS Catal. 1 (6), 657–664. 10.1021/cs200105u

[B70] Li F. Frett B. Li H.-y. (2014). Selective reduction of halogenated nitroarenes with hydrazine hydrate in the presence of Pd/C. Synlett 25 (10), 1403–1408. 10.1055/s-0033-1339025 26843785 PMC4734645

[B71] Li H. Sun Z. Fan Y. Zhang G. Ni S. Q. Gawande M. B. (2025a). Enhancing hydride formation and transfer for catalytic hydrogenation *via* electron-deficient single-atom silver. J. Colloid Interface Sci. 682, 751–759. 10.1016/j.jcis.2024.11.223 39644746

[B72] Li S. Millan M. Gao F. Long X. Yuan G. Cui Z. (2025b). Synergistic effect of Ru-Ni nanoparticles supported on mesoporous carbon black for highly selective hydrogenation of nitrobenzene to aniline in aqueous phase. Fuel 402, 136060. 10.1016/j.fuel.2025.136060

[B73] Liao H. Weng P. Huang H. Tan R. Zhu R. Liu Y. (2023). Ultrafine Ru nanoparticles integrated on ordered mesoporous carbon for solvent-free hydrogenation of nitroarenes. RSC Adv. 13 (30), 20876–20888. 10.1039/d3ra03643j 37448646 PMC10336478

[B74] Liu Y. Miao W. Tang W. Xue D. Xiao J. Wang C. (2021a). Rhodium‐terpyridine catalyzed transfer hydrogenation of aromatic nitro compounds in water. Chemistry–An Asian J. 16 (13), 1725–1729. 10.1002/asia.202100321 33950565

[B75] Liu Y. Zhang W. Zheng Y. Wu K. Dong P. He R. (2021b). Single-atom Fe–N 4 site for the hydrogenation of nitrobenzene: theoretical and experimental studies. Dalton Trans. 50 (23), 7995–8001. 10.1039/d1dt01227d 34019047

[B76] Liu S. Amaro-Estrada J. I. Baltrun M. Douair I. Schoch R. Maron L. (2021c). Catalytic deoxygenation of nitroarenes mediated by high-valent molybdenum (VI)–NHC complexes. Organometallics 40 (2), 107–118. 10.1021/acs.organomet.0c00352

[B77] Liu Z. Wang W. Zhao Y. Jing Z. Wan R. Li H. (2022a). Synthesis, structure, and catalytic activities of two multi-rh-decorated polyoxometalates. Inorg. Chem. 61 (39), 15310–15314. 10.1021/acs.inorgchem.2c02220 36129305

[B78] Liu W. Feng H. Yang Y. Niu Y. Wang L. Yin P. (2022b). Highly-efficient RuNi single-atom alloy catalysts toward chemoselective hydrogenation of nitroarenes. Nat. Commun. 13 (1), 3188. 10.1038/s41467-022-30536-9 35676245 PMC9178046

[B79] Liu X. Wang C. Meng J. Yue X. Wang Q. Lu J. (2023). Single-atom cobalt catalysts for chemoselective hydrogenation of nitroarenes to anilines. Chin. Chem. Lett. 34 (12), 108745. 10.1016/j.cclet.2023.108745

[B80] Liu M. Gong X. Yang Y. Yu Y. Song X. Xie K. (2025). Modulation of p–d orbital hybridization in Pt-M (p-block) dual-atom catalysts to promote the activity of electrocatalytic nitrobenzene reduction. Appl. Surf. Sci. 716, 164657. 10.1016/j.apsusc.2025.164657

[B81] Loos P. Alex H. Hassfeld J. Lovis K. Platzek J. Steinfeldt N. (2016). Selective hydrogenation of halogenated nitroaromatics to haloanilines in batch and flow. Org. Process Res. Dev. 20 (2), 452–464. 10.1021/acs.oprd.5b00170

[B82] Lu X. Qin J. Xian C. Nie J. Li X. He J. (2022). Cobalt nanoparticles supported on microporous nitrogen-doped carbon for an efficient catalytic transfer hydrogenation reaction between nitroarenes and N-heterocycles. Catal. Sci. Technol. 12 (18), 5549–5558. 10.1039/d2cy00914e

[B83] Lu A. Xiang X. Lei M. Huang S. Liang B. Zhao S. (2025). Highly efficient catalytic transfer hydrogenation for the conversion of nitrobenzene to aniline over PdO/TiO_2_: the key role of *in situ* switching from PdO to Pd. J. Environ. Sci. 148, 515–528. 10.1016/j.jes.2023.10.010 39095185

[B84] Luo P. Xu K. Zhang R. Huang L. Wang J. Xing W. (2012). Highly efficient and selective reduction of nitroarenes with hydrazine over supported rhodium nanoparticles. Catal. Sci. Technol. 2 (2), 301–304. 10.1039/c1cy00358e

[B85] Luo S. Long Y. Liang K. Qin J. Qiao Y. Li J. (2021). Unsaturated Mo in Mo_4_O_4_N_3_ for efficient catalytic transfer hydrogenation of nitrobenzene using stoichiometric hydrazine hydrate. Green Chem. 23 (21), 8545–8553. 10.1039/d1gc02647j

[B86] Luo X. Fu C. Shen S. Luo L. Zhang J. (2023). Free–templated synthesis of N–doped PtCu porous hollow nanospheres for efficient ethanol oxidation and oxygen reduction reactions. Appl. Catal. B Environ. 330, 122602. 10.1016/j.apcatb.2023.122602

[B87] Ma L. Chen P. Zhang G. Wang L. Tang F. Zhao X. (2021). Promoting H2 activation over Molybdenum Carbide by modulation of metal‐support interaction for efficient catalytic hydrogenation. ChemCatChem 13 (14), 3283–3289. 10.1002/cctc.202100581

[B88] Mahajan A. Gupta M. (2021). Hybrid ceria and chitosan supported nickel nanoparticles: a recyclable nanocatalytic system in the reduction of nitroarenes and the synthesis of benzopyran derivatives in green solvent. Appl. Organomet. Chem. 35 (5), e6161. 10.1002/aoc.6161

[B89] Mahata N. Cunha A. Orfao J. Figueiredo J. (2008). Hydrogenation of nitrobenzene over nickel nanoparticles stabilized by filamentous carbon. Appl. Catalysis A General 351 (2), 204–209. 10.1016/j.apcata.2008.09.015

[B90] Manikandan R. Shanmugam R. Pratheepkumar A. (2025). Engaging hydrazine hydrate as a hydrogen source for cobalt (ii)-catalysed transfer hydrogenation of nitroaromatics. Chem. Commun. 61, 9043–9046. 10.1039/D5CC01160D 40465558

[B91] Mao Q. Mu X. Wang W. Deng K. Yu H. Wang Z. (2023). Atomically dispersed Cu coordinated Rh metallene arrays for simultaneously electrochemical aniline synthesis and biomass upgrading. Nat. Commun. 14 (1), 5679. 10.1038/s41467-023-41423-2 37709775 PMC10502102

[B92] Martina K. Moran M. J. Manzoli M. Trukhan M. V. Kuhn S. Van Gerven T. (2023). Copper-Catalyzed continuous-flow transfer hydrogenation of nitroarenes to anilines: a scalable and reliable protocol. Org. Process Res. Dev. 28 (5), 1515–1528. 10.1021/acs.oprd.3c00144 38783856 PMC11110069

[B93] Maurya A. Patel U. K. Kumar S. Agarwal A. (2024). Molybdenum trioxide as a newer diversified economic catalyst for the transformation of nitroarenes to arylamine and 5-substituted-1 H-tetrazole. RSC Adv. 14 (40), 29505–29517. 10.1039/d4ra05443a 39297051 PMC11409451

[B94] McCullagh A. Davidson A. Ballas C. How C. MacLaren D. Boulho C. (2024). The application of an alumina-supported Ni catalyst for the hydrogenation of nitrobenzene to aniline. Catal. Today 442, 114933. 10.1016/j.cattod.2024.114933

[B95] McManus I. J. Daly H. Manyar H. G. Taylor S. F. R. Thompson J. M. Hardacre C. (2016). Selective hydrogenation of halogenated arenes using porous manganese oxide (OMS-2) and platinum supported OMS-2 catalysts. Faraday Discuss. 188, 451–466. 10.1039/c5fd00227c 27095631

[B96] Meng X. Cheng H. Fujita S. i. Yu Y. Zhao F. Arai M. (2011). An effective medium of H_2_O and low-pressure CO_2_ for the selective hydrogenation of aromatic nitro compounds to anilines. Green Chem. 13 (3), 570–572. 10.1039/c0gc00246a

[B97] Mirhosseyni M. S. Nemati F. Nahzomi H. T. (2021). Incorporation of copper nanoparticles into the nitrogen‐doped carbon derived from nitrile functionalized ionic liquid as the non‐precious heterogeneous catalytic system toward nitro compounds reduction reaction, a first principle calculation. J. Chem. Technol. Biotechnol. 96 (10), 2802–2812. 10.1002/jctb.6827

[B98] Mitsudome T. Kaneda K. (2013). Gold nanoparticle catalysts for selective hydrogenations. Green Chem. 15 (10), 2636–2654. 10.1039/c3gc41360h

[B99] Moran M. J. Martina K. Stefanidis G. D. Jordens J. Gerven T. V. Goovaerts V. (2020a). Glycerol: an optimal hydrogen source for microwave-promoted Cu-catalyzed transfer hydrogenation of nitrobenzene to aniline. Front. Chem. 8, 34. 10.3389/fchem.2020.00034 32064251 PMC7000456

[B100] Moran M. J. Martina K. Baricco F. Tagliapietra S. Manzoli M. Cravotto G. (2020b). Tuneable copper catalysed transfer hydrogenation of nitrobenzenes to aniline or azo derivatives. Adv. Synthesis Catal. 362 (13), 2689–2700. 10.1002/adsc.202000127

[B101] Morisse C. G. McCullagh A. M. Campbell J. W. How C. MacLaren D. A. Carr R. H. (2021). Toward high selectivity aniline synthesis catalysis at elevated temperatures. Industrial Eng. Chem. Res. 60 (49), 17917–17927. 10.1021/acs.iecr.1c03695 PMC8802303 35115738

[B102] Nagy C. Sikora E. Prekob Á. Gráczer K. Muránszky G. Vanyorek L. (2025). Spherified Pd0. 33Ni0. 67/BCNT catalyst for nitrobenzene hydrogenation. Int. J. Mol. Sci. 26 (11), 5420. 10.3390/ijms26115420 40508227 PMC12155155

[B103] Nandi D. Siwal S. Mallick K. (2017). A carbon nitride supported copper nanoparticle composite: a heterogeneous catalyst for the N-arylation of hetero-aromatic compounds. New J. Chem. 41 (8), 3082–3088. 10.1039/c6nj03584a

[B104] Nie R. Wang J. Wang L. Qin Y. Chen P. Hou Z. (2012). Platinum supported on reduced graphene oxide as a catalyst for hydrogenation of nitroarenes. Carbon 50 (2), 586–596. 10.1016/j.carbon.2011.09.017

[B105] Nie S. Yang S. Zhang P. (2020). Solvent-free synthesis of mesoporous platinum-aluminum oxide *via* mechanochemistry: toward selective hydrogenation of nitrobenzene to aniline. Chem. Eng. Sci. 220, 115619. 10.1016/j.ces.2020.115619

[B106] Nieto-Márquez A. Gil S. Romero A. Valverde J. L. Gómez-Quero S. Keane M. A. (2009). Gas phase hydrogenation of nitrobenzene over acid treated structured and amorphous carbon supported Ni catalysts. Appl. Catal. A General 363 (1-2), 188–198. 10.1016/j.apcata.2009.05.016

[B107] Nuzhdin A. L. Bukhtiyarova M. V. Bukhtiyarova G. A. (2024). Organic synthesis in flow mode by selective liquid-phase hydrogenation over heterogeneous non-noble metal catalysts. Org. Biomol. Chem. 22, 7936–7950. 10.1039/D4OB00873A 39254682

[B108] Pal D. Pal A. (2025). Ammonium phosphomolybdate–titanium dioxide composite material as a catalyst for antibiotic degradation under ambient conditions. Catal. Sci. Technol. 15, 4945–4963. 10.1039/D5CY00234F

[B109] Park J. Joo J. Kwon S. G. Jang Y. Hyeon T. (2007). Synthesis of monodisperse spherical nanocrystals. Angew. Chem. Int. Ed. 46 (25), 4630–4660. 10.1002/anie.200603148 17525914

[B110] Park J. Evans C. Maier J. Hatzell M. France S. Sievers C. (2024). Renewables-Based routes to paracetamol: a green chemistry analysis. ACS Sustain. Chem. Eng. 12 (44), 16271–16282. 10.1021/acssuschemeng.4c05353

[B111] Paterson R. Alharbi H. Wills C. Chamberlain T. Bourne R. A. Griffiths A. (2022). Highly efficient and selective reduction of nitroarenes to N-Arylhydroxylamines catalysed by Phosphine oxide-decorated polymer immobilized ionic liquid stabilized ruthenium nanoparticles.

[B112] Paterson R. Alharbi H. Y. Wills C. Chamberlain T. W. Bourne R. A. Griffiths A. (2023). Highly efficient and selective partial reduction of nitroarenes to N-arylhydroxylamines catalysed by phosphine oxide-decorated polymer immobilized ionic liquid stabilized ruthenium nanoparticles. J. Catal. 417, 74–88. 10.1016/j.jcat.2022.11.023

[B113] Patil U. D. Vinchurkar A. N. Narkhede J. S. Bari M. L. Deshpande T. D. (2025). Navigating heterogeneous hydrogenation hazards: a systematic approach from primary screening to catalyst loading and optimization parameters. Chem. Pap. 79 (3), 1637–1647. 10.1007/s11696-024-03879-4

[B114] Patzsch J. Berg B. Bloh J. Z. (2019). Kinetics and optimization of the photocatalytic reduction of nitrobenzene. Front. Chem. 7, 289. 10.3389/fchem.2019.00289 31069220 PMC6491869

[B115] Paul R. Ahamed S. S. Ghosh T. (2025). Nickel‐Catalyzed hydrogenation and dehydrogenation processes: a useful tool in organic synthesis. Asian J. Org. Chem. 14 (9), e00069. 10.1002/ajoc.202500069

[B116] Pedrajas E. Sorribes I. Junge K. Beller M. Llusar R. (2017). Selective reductive amination of aldehydes from nitro compounds catalyzed by molybdenum sulfide clusters. Green Chem. 19 (16), 3764–3768. 10.1039/c7gc01603d

[B117] Petrelli V. Romanazzi G. Mortalò C. Leonelli C. Zapparoli M. De Giglio E. (2023). N-doped resin supported cobalt nanoparticles for the catalytic reduction of nitroarenes to corresponding anilines in aqueous medium. Mol. Catal. 544, 113050. 10.1016/j.mcat.2023.113050

[B118] Petrov L. Kirkov N. Shopov D. (1986). Kinetics of hydrogenation of nitrobenzene to aniline on a copper catalyst. Kinet. Catal. (Engl. Transl.) 26 (4). Available online at: https://www.osti.gov/biblio/7005047 .

[B119] Petrov L. Kumbilieva K. Kirkov N. (1990). Kinetic model of nitrobenzene hydrogenation to aniline over industrial copper catalyst considering the effects of mass transfer and deactivation. Appl. Catalysis 59 (1), 31–43. 10.1016/s0166-9834(00)82185-5

[B120] Phasayavan W. Inceesungvorn B. Chansai S. Hardacre C. (2024). MOO3 catalysed hydrogenation of nitrobenzene to aniline at near room temperature. Inorg. Chem. Commun. 164, 112416. 10.1016/j.inoche.2024.112416

[B121] Qi H. Wang X. Lei M. Fan W. Huang S. Zhu L. (2024). Highly efficient catalytic hydrogenation of nitrobenzene on cobalt-immobilized nitrogen-doped carbon: a dual-sites synergistic effect between cobalt single atoms and cobalt nanoparticles. Chem. Eng. J. 500, 157057. 10.1016/j.cej.2024.157057

[B122] Qu Y. Yang H. Wang S. Chen T. Wang G. (2017). Hydrogenation of nitrobenzene to aniline catalyzed by C60-stabilized Ni. Catal. Commun. 97, 83–87. 10.1016/j.catcom.2017.04.029

[B123] Qusti A. Mohamed R. Salam M. A. (2014). Photocatalytic synthesis of aniline from nitrobenzene using Ag-reduced graphene oxide nanocomposite. Ceram. Int. 40 (4), 5539–5546. 10.1016/j.ceramint.2013.10.144

[B124] Rai R. K. Mahata A. Mukhopadhyay S. Gupta S. Li P. Z. Nguyen K. T. (2014). Room-temperature chemoselective reduction of nitro groups using non-noble metal nanocatalysts in water. Inorg. Chem. 53 (6), 2904–2909. 10.1021/ic402674z 24564248

[B125] Raimundo B. Kino D. Kitgawa N. Tokudome Y. Nunes C. D. (2023). Co-oxide nanostructured catalysts tailored from layered double hydroxides for highly efficient hydrogenation of nitroarenes. Appl. Clay Sci. 239, 106948. 10.1016/j.clay.2023.106948

[B126] Raj K. J. A. Prakash M. Mahalakshmy R. Elangovan T. Viswanathan B. (2012). Liquid phase hydrogenation of nitrobenzene over nickel supported on titania. Chin. J. Catal. 33 (7-8), 1299–1305. 10.1016/s1872-2067(11)60398-7

[B127] Rana S. Varadwaj G. B. B. Jonnalagadda S. B. (2021). Manganese oxide supported partially reduced graphene oxide as a highly active and durable catalyst for the amination of benzene. Catal. Commun. 157, 106329. 10.1016/j.catcom.2021.106329

[B128] Rana A. Yadav M. Kumar R. Singh B. Meena Y. S. Naik G. (2025). Chemoselective hydrogenation of nitroarenes to arylamines using manganese nanocatalyst and molecular hydrogen. ACS Appl. Nano Mater. 8 (49), 23357–23369. 10.1021/acsanm.5c03553

[B129] Rohmah A. i.N. Palupi A. R. Manullang A. N. H. M. Erawati H. D. B. (2025). Aniline process creation for conversion improvement using hydrogenation process. J. Chem. Eng. Res. Prog. 2 (1), 132–141. 10.9767/jcerp.20292

[B130] Rostami S. Yahyazadeh A. Adibi H. (2024). Designing a new magnetic g-C3N4 nanocatalyst based on Ag nanoparticles supported by β-cyclodextrin for effective reduction of nitroaromatic compounds. Sci. Rep. 14 (1), 31586. 10.1038/s41598-024-76786-z 39738240 PMC11686344

[B131] Ruan L. Han X. Zhu L. Shang C. Kroner A. Liu K. (2025). Rhodium nanoparticles in ZrO_2_ on N-doped carbon leads to ultra-high catalytic selectivity and activity in nitroarene hydrogenation. Appl. Catal. B Environ. Energy 379, 125686. 10.1016/j.apcatb.2025.125686

[B132] Rubab A. Sharif M. Razzaq R. Jackstell R. Nafady A. Weiß J. (2024). Highly active iron oxide@ N catalyst derived from the iron acetate/polyacrylonitrile threads: driving nitroarene conversion to value‐added amines. J. Appl. Polym. Sci. 141 (41), e56062. 10.1002/app.56062

[B133] Rubio-Marqués P. Leyva-Pérez A. Corma A. (2013). A bifunctional palladium/acid solid catalyst performs the direct synthesis of cyclohexylanilines and dicyclohexylamines from nitrobenzenes. Chem. Commun. 49 (74), 8160–8162. 10.1039/c3cc44064h 23925659

[B134] Rueping M. Dufour J. Schoepke F. R. (2011). Advances in catalytic metal-free reductions: from bio-inspired concepts to applications in the organocatalytic synthesis of pharmaceuticals and natural products. Green Chem. 13 (5), 1084–1105. 10.1039/c1gc15027h

[B135] Saha S. Pal A. Kundu S. Basu S. Pal T. (2010). Photochemical green synthesis of calcium-alginate-stabilized Ag and Au nanoparticles and their catalytic application to 4-nitrophenol reduction. Langmuir 26 (4), 2885–2893. 10.1021/la902950x 19957940

[B136] Shah M. Guo Q.-X. Fu Y. (2015). The colloidal synthesis of unsupported nickel‐tin bimetallic nanoparticles with tunable composition that have high activity for the reduction of nitroarenes. Catal. Commun. 65, 85–90. 10.1016/j.catcom.2015.02.026

[B137] Shen Y. Zhu C. Chen B. (2020). Immobilizing 1–3 nm Ag nanoparticles in reduced graphene oxide aerogel as a high-effective catalyst for reduction of nitroaromatic compounds. Environ. Pollut. 256, 113405. 10.1016/j.envpol.2019.113405 31672347

[B138] Sheng Y. Liu Y. Yin Y. Zou X. Ren J. Wu B. (2023). Rh promotional effects on Pt–Rh alloy catalysts for chemoselective hydrogenation of nitrobenzene to p-aminophenol. Chem. Eng. J. 452, 139448. 10.1016/j.cej.2022.139448

[B139] Shimizu K.-i. Miyamoto Y. Satsuma A. (2010). Size-and support-dependent silver cluster catalysis for chemoselective hydrogenation of nitroaromatics. J. Catal. 270 (1), 86–94. 10.1016/j.jcat.2009.12.009

[B140] Shokouhimehr M. (2015). Magnetically separable and sustainable nanostructured catalysts for heterogeneous reduction of nitroaromatics. Catalysts 5 (2), 534–560. 10.3390/catal5020534

[B141] Singha Hazari A. Frisch M. L. Wen Y. Stankovic M. D. Berlinguette C. P. (2024). Electrolytic conversion of nitro compounds into amines in a membrane reactor. J. Am. Chem. Soc. 146 (41), 28153–28160. 10.1021/jacs.4c07847 39353136

[B142] Sokolova D. Lurshay T. C. Rowbotham J. S. Stonadge G. Reeve H. A. Cleary S. E. (2024). Selective hydrogenation of nitro compounds to amines by coupled redox reactions over a heterogeneous biocatalyst. Nat. Commun. 15 (1), 7297. 10.1038/s41467-024-51531-2 39181899 PMC11344822

[B143] Soni Y. Philip M. C. Vinod C. P. (2025). Ultra-Small Pd nanoparticles on SBA-15: an efficient catalyst for one-pot reductive alkylation of nitrobenzene with size-dependent activity. Top. Catal. 68 (3), 414–429. 10.1007/s11244-024-02042-3

[B144] Southouse J. P. Lazzarini L. Ibhadon A. O. Francesconi M. G. (2021). Ultra-small FeS 2 nanoparticles for highly efficient chemoselective transfer hydrogenation of nitroarenes. New J. Chem. 45 (38), 17808–17815. 10.1039/d1nj03297f

[B145] Sreedhar B. Devi D. K. Yada D. (2011). Selective hydrogenation of nitroarenes using gum acacia supported Pt colloid an effective reusable catalyst in aqueous medium. Catal. Commun. 12 (11), 1009–1014. 10.1016/j.catcom.2011.02.027

[B146] Stergiou A. D. Broadhurst D. H. Symes M. D. (2022). Highly selective electrocatalytic reduction of substituted nitrobenzenes to their aniline derivatives using a polyoxometalate redox mediator. ACS Org. Inorg. Au 3 (1), 51–58. 10.1021/acsorginorgau.2c00047 36748077 PMC9896480

[B147] Stiles A. B. (1987). Catalyst supports and supported catalysts.

[B148] Su X. Kanjanawarut R. (2009). Control of metal nanoparticles aggregation and dispersion by PNA and PNA− DNA complexes, and its application for colorimetric DNA detection. Acs Nano 3 (9), 2751–2759. 10.1021/nn9005768 19708641

[B149] Sun Z. Zhao Y. Xie Y. Tao R. Zhang H. Huang C. (2010). The solvent-free selective hydrogenation of nitrobenzene to aniline: an unexpected catalytic activity of ultrafine Pt nanoparticles deposited on carbon nanotubes. Green Chem. 12 (6), 1007–1011. 10.1039/c002391d

[B150] Taheri-Ledari R. Mirmohammadi S. S. Valadi K. Maleki A. Shalan A. E. (2020). Convenient conversion of hazardous nitrobenzene derivatives to aniline analogues by Ag nanoparticles, stabilized on a naturally magnetic pumice/chitosan substrate. RSC Adv. 10 (71), 43670–43681. 10.1039/d0ra08376c 35519713 PMC9058380

[B151] Tan E. Montesinos-Magraner M. García-Morales C. Mayans J. G. Echavarren A. M. (2021). Rhodium-catalysed ortho-alkynylation of nitroarenes. Chem. Sci. 12 (44), 14731–14739. 10.1039/d1sc04527j 34820088 PMC8597868

[B152] Tang J. Zhang S. Chen X. Zhang L. Du L. Zhao Q. (2023). Highly efficient catalytic reduction of nitrobenzene using Cu@ C based on a novel Cu–MOF precursor. Catalysts 13 (6), 956. 10.3390/catal13060956

[B153] Tang W. Liu Y. Jin Y. Wang Y. Shi W. Ma P. (2024). Photocatalytic reduction of nitrobenzene to aniline by an intriguing {Ru (C6H6)}-based heteropolytungstate. Inorg. Chem. 63 (14), 6260–6267. 10.1021/acs.inorgchem.3c04450 38517738

[B154] Tavakolian M. Saki S. Hosseini-Sarvari M. (2025). Selective photocatalytic reduction of nitrobenzene to anilines, azoxybenzene, and azobenzene: a solvent-dependent and light-induced process mediated by a CdS/NH 2-MIL-125 nanocomposite. Org. Biomol. Chem. 23 (27), 6625–6636. 10.1039/d5ob00705d 40575996

[B155] Tian S. Hu M. Xu Q. Gong W. Chen W. Yang J. (2021). Single-atom Fe with Fe1N3 structure showing superior performances for both hydrogenation and transfer hydrogenation of nitrobenzene. Sci. China Mater. 64 (3), 642–650. 10.1007/s40843-020-1443-8

[B156] Timelthaler D. Schöfberger W. Topf C. (2021). Selective and additive‐free hydrogenation of nitroarenes mediated by a DMSO‐tagged molecular cobalt corrole catalyst. Eur. J. Org. Chem. 2021 (14), 2114–2120. 10.1002/ejoc.202100073 PMC8252576 34248412

[B157] Torabi S. Nasiriani T. Javanbakht S. Shaabani A. (2025). Anchoring silver nanoparticles on graphene quantum dots: a highly efficient, green, and rapid nano-catalyst for the reduction of nitro compounds and tandem reductive Ugi reactions. J. Phys. Chem. Solids 201, 112633. 10.1016/j.jpcs.2025.112633

[B158] Toshima N. Wang Y. (1994). Preparation and catalysis of novel colloidal dispersions of copper/noble metal bimetallic clusters. Langmuir 10 (12), 4574–4580. 10.1021/la00024a031

[B159] Trasarti A. F. Bertero N. M. Apesteguia C. R. Marchi A. J. (2014). Liquid-phase hydrogenation of acetophenone over silica-supported Ni, Co and Cu catalysts: influence of metal and solvent. Appl. Catal. A General 475, 282–291. 10.1016/j.apcata.2014.01.038

[B160] Turáková M. Salmi T. Eränen K. Wärnå J. Murzin D. Y. Králik M. (2015). Liquid phase hydrogenation of nitrobenzene. Appl. Catal. A General 499, 66–76. 10.1016/j.apcata.2015.04.002

[B161] Unglaube F. Kreyenschulte C. R. Mejía E. (2021). Development and application of efficient ag‐based hydrogenation catalysts prepared from Rice Husk waste. ChemCatChem 13 (11), 2583–2591. 10.1002/cctc.202100045

[B162] Unglaube F. Schlapp J. Quade A. Schäfer J. Mejía E. (2022). Highly active heterogeneous hydrogenation catalysts prepared from cobalt complexes and rice husk waste. Catal. Sci. Technol. 12 (10), 3123–3136. 10.1039/d2cy00005a

[B163] Vengatesh G. Nanjan P. (2022). Reduction of electron-rich nitro heteroarenes; a comprehensive review. Curr. Org. Chem. 26 (17), 1626–1637. 10.2174/1385272827666221128113437

[B164] Wang W. Zhang L. (2014). Hydrogen transfer hydrogenation of nitrobenzene to aniline with Ru (acac) 3 as the catalyst. Res. Chem. Intermed. 40 (8), 3109–3118. 10.1007/s11164-013-1155-7

[B165] Wang A.-J. Cheng H. Y. Liang B. Ren N. Q. Cui D. Lin N. (2011). Efficient reduction of nitrobenzene to aniline with a biocatalyzed cathode. Environ. Sci. Technol. 45 (23), 10186–10193. 10.1021/es202356w 21985580

[B166] Wang X. Yan C. Sumboja A. Lee P. S. (2014). Nickel cobalt oxide nanowire-reduced graphite oxide composite material and its application for high performance supercapacitor electrode material. J. Nanosci. Nanotechnol. 14 (9), 7104–7110. 10.1166/jnn.2014.8982 25924377

[B167] Wang X. Perret N. Delannoy L. Louis C. Keane M. A. (2016). Selective gas phase hydrogenation of nitroarenes over Mo 2 C-supported Au–Pd. Catal. Sci. Technol. 6 (18), 6932–6941. 10.1039/c6cy00514d

[B168] Wang H. Zhang W. Liu Y. Pu M. Lei M. (2021). First-principles study on the mechanism of nitrobenzene reduction to aniline catalyzed by a N-doped carbon-supported cobalt single-atom catalyst. J. Phys. Chem. C 125 (35), 19171–19182. 10.1021/acs.jpcc.1c01877

[B169] Wang H. Pu M. Lei M. (2025). Theoretical study on nitrobenzene hydrogenation to aniline catalyzed by M 1/CeO 2− x (111) single-atom catalysts. Phys. Chem. Chem. Phys. 27 (9), 4829–4836. 10.1039/d4cp04459b 39957625

[B170] Wong A.J.-W. Miller J. L. Janik M. J. (2022). Elementary mechanism for the electrocatalytic reduction of nitrobenzene on late-transition-metal surfaces from density functional theory. Chem. Catal. 2 (6), 1362–1379. 10.1016/j.checat.2022.03.009

[B171] Wu J. Darcel C. (2023). Recent developments in manganese, iron and cobalt homogeneous catalyzed synthesis of primary amines *via* reduction of nitroarenes, nitriles and carboxamides. Adv. Synthesis Catal. 365 (7), 948–964. 10.1002/adsc.202201346

[B172] Wu S. Wen G. Schlögl R. Su D. S. (2015). Carbon nanotubes oxidized by a green method as efficient metal-free catalysts for nitroarene reduction. Phys. Chem. Chem. Phys. 17 (3), 1567–1571. 10.1039/c4cp04658g 25474718

[B173] Xie Y. Shang X. Liu D. Zhao H. Gu Y. Zhang Z. (2019). Non-noble metal thickness-tunable Bi_2_MoO_6_ nanosheets for highly efficient visible-light-driven nitrobenzene reduction into aniline. Appl. Catal. B Environ. 259, 118087. 10.1016/j.apcatb.2019.118087

[B174] Xu L. Kuang H. Xu C. Ma W. Wang L. Kotov N. A. (2012). Regiospecific plasmonic assemblies for *in situ* Raman spectroscopy in live cells. J. Am. Chem. Soc. 134 (3), 1699–1709. 10.1021/ja2088713 22192084 PMC3277787

[B175] Xu S.-S. Liang J. Z. Yao S. J. Li N. Shi J. W. Lan Y. Q. (2026). Microenvironment regulation of active sites for efficient photocatalytic reduction of nitrobenzene. Chem. Commun. 10.1039/D5CC04423E 41369643

[B176] Yadav G. D. Mewada R. K. (2013). Novelties of azobenzene synthesis *via* selective hydrogenation of nitrobenzene over nano-fibrous Ag-OMS-2–Mechanism and kinetics. Chem. Eng. J. 221, 500–511. 10.1016/j.cej.2013.01.074

[B177] Yamaguchi S. Sarmiento-Diaz M. Kawakami T. Tada K. Mitsudome T. Mizugaki T. (2025). Efficient hydrogenation of nitrobenzene to aniline over a palladium Carbide nanocube catalyst. ACS Appl. Nano Mater. 8 (27), 13886–13893. 10.1021/acsanm.5c02582

[B178] Yánez J. E. Rivas A. B. Alvarez J. Ortega M. C. Pardey A. J. Longo C. (2006). Reduction of nitrobenzene catalyzed by immobilized copper catalyst under carbon monoxide and water. J. Coord. Chem. 59 (15), 1719–1728. 10.1080/00958970600586347

[B179] Yang Q. Chen Y. Z. Wang Z. U. Xu Q. Jiang H. L. (2015a). One-pot tandem catalysis over Pd@ MIL-101: boosting the efficiency of nitro compound hydrogenation by coupling with ammonia borane dehydrogenation. Chem. Commun. 51 (52), 10419–10422. 10.1039/c5cc03102h 26000763

[B180] Yang H. Cui X. Dai X. Deng Y. Shi F. (2015b). Carbon-catalysed reductive hydrogen atom transfer reactions. Nat. Commun. 6 (1), 6478. 10.1038/ncomms7478 25832812

[B181] Yao J. Wang D. Kongr D. Chu M. Wang J. Liu L. (2025). Aqueous-Phase reduction of Nitrobenzene enabled by a symmetry-disrupted Fe single-atom catalyst featuring phosphorus coordination. ACS Appl. Mater. Interfaces. 17 (43), 59422–59433. 10.1021/acsami.5c15977 41108652

[B182] Zarei H. Sobhani S. Sansano J. M. (2024). An innovative water splitting process for a sustainable *in situ* hydrogen generation–reduction of nitrobenzenes catalyzed by a new Bimetallic nanocatalyst. Appl. Organomet. Chem. 38 (12), e7740. 10.1002/aoc.7740

[B183] Zhang Y. Liu S. Lu W. Wang L. Tian J. Sun X. (2011a). *In situ* green synthesis of Au nanostructures on graphene oxide and their application for catalytic reduction of 4-nitrophenol. Catal. Sci. Technol. 1 (7), 1142–1144. 10.1039/c1cy00205h

[B184] Zhang F. Jin J. Zhong X. Li S. Niu J. Li R. (2011b). Pd immobilized on amine-functionalized magnetite nanoparticles: a novel and highly active catalyst for hydrogenation and Heck reactions. Green Chem. 13 (5), 1238–1243. 10.1039/c0gc00854k

[B185] Zhang L. Shao Z.-J. Cao X.-M. Hu P. (2018). Insights into different products of nitrosobenzene and nitrobenzene hydrogenation on Pd (111) under realistic reaction conditions. J. Phys. Chem. C 122 (35), 20337–20350. 10.1021/acs.jpcc.8b05364

[B186] Zhang L. Zhao R. Li H. Bao J. Wang Q. (2023a). Promotional effects of phosphotungstic acid on CePO_4_ catalyst for the selective catalytic reduction of NOx with NH3. Mol. Catal. 551, 113666. 10.1016/j.mcat.2023.113666

[B187] Zhang Y. Li Z. Zhang J. Xu L. Han Z. K. Baiker A. (2023b). Nanostructured Ni-MoC x: an efficient non-noble metal catalyst for the chemoselective hydrogenation of nitroaromatics. Nano Res. 16 (7), 8919–8928. 10.1007/s12274-023-5598-x

[B188] Zhang K. Yu J. Ru C. Mu L. Li J. Shi W. (2024). Photoelectrocatalytic Reduction of Nitrobenzene to Azobenzene by Using Ag Nanoparticles‐Decorated Si Nanocone Arrays Photocathodes. ChemPhotoChem 8 (11), e202400099. 10.1002/cptc.202400099

[B189] Zhang S. Sun Y. Chen W. Li H. Li K. Ma P. (2025a). Facile synthesis of a Ru-based polyoxometalate for efficient reduction of nitrobenzene. Dalton Trans. 54 (23), 9352–9358. 10.1039/D5DT00020C 40407012

[B190] Zhang C. Chabu J. M. Wang L. Liu Y.-N. (2025b). Construction of Ni− Ov− Ti sites through strong metal-support interactions for selective hydrogenation of nitroarenes to anilines. Chem. Eng. J. 521, 166785. 10.1016/j.cej.2025.166785

[B191] Zhang Y.-X. Ma Y. J. Fei H. M. Zhang F. Lan L. J. Yu C. X. (2026). Efficient and selective catalytic hydrogenation of nitroarenes catalyzed by two-dimensional ni@ cu-MOF bimetallic nanosheets. Sep. Purif. Technol. 388, 136830. 10.1016/j.seppur.2026.136830

[B192] Zhao Z. Yang H. Li Y. Guo X. (2014). Cobalt-modified molybdenum carbide as an efficient catalyst for chemoselective reduction of aromatic nitro compounds. Green Chemistry 16 (3), 1274–1281. 10.1039/c3gc42049c

[B193] Zhao B. Dong Z. Wang Q. Xu Y. Zhang N. Liu W. (2020). Highly efficient mesoporous core-shell structured ag@ SiO_2_ nanosphere as an environmentally friendly catalyst for hydrogenation of nitrobenzene. Nanomaterials 10 (5), 883. 10.3390/nano10050883 32375276 PMC7279246

[B194] Zhao C. C. Wang S. Yan L. K. Su Z. M. (2024). Insights into the mechanism of nitrobenzene reduction to aniline by phosphomolybdic acid supported TM1 single-atom catalysts. Inorg. Chem. 63 (4), 1784–1792. 10.1021/acs.inorgchem.3c03106 38232070

[B195] Zheng C. You S.-L. (2012). Transfer hydrogenation with Hantzsch esters and related organic hydride donors. Chem. Soc. Rev. 41 (6), 2498–2518. 10.1039/c1cs15268h 22282764

[B196] Zhou P. Jiang L. Wang F. Deng K. Lv K. Zhang Z. (2017). High performance of a cobalt–nitrogen complex for the reduction and reductive coupling of nitro compounds into amines and their derivatives. Sci. Adv. 3 (2), e1601945. 10.1126/sciadv.1601945 28232954 PMC5315448

[B197] Zhou W. Xie X. Yang C. Zhang T. Yan X. Hu X. (2024). Adsorption-Dependent H_2_O effect for nitrobenzene hydrogenation to aniline over Pt and Ni catalysts. Industrial Eng. Chem. Res. 63 (31), 13536–13543. 10.1021/acs.iecr.4c01884

[B198] Zhou T. Yang Y. Du T. Dong W. Wang H. (2025). “Degradation kinetics of Nitrobenzene and aniline by Fe-N/BC Redox System,” in Journal of physics: conference series (New York, United States: IOP Publishing), 2941 (1), 012027.

[B199] Zirak M. Jalalat M. Vahdati-Khajeh S. Gargari M. S. Rad S. L. Eftekhari-Sis B. (2025). Biomass-derived N-doped ordered mesoporous carbon-supported gold nanoparticles: an efficient catalyst for the reduction of nitroaromatic pollutants. J. Inorg. Organomet. Polym. Mater. 35 (1), 527–537. 10.1007/s10904-024-03307-w

[B200] Zou K. Guan Z. Deng Y. Chen G. (2020). Nitrogen-rich porous carbon in ultra-high yield derived from activation of biomass waste by a novel eutectic salt for high performance Li-ion capacitors. Carbon 161, 25–35. 10.1016/j.carbon.2020.01.045

[B201] Zubar V. Dewanji A. Rueping M. (2021). Chemoselective hydrogenation of nitroarenes using an air-stable base-metal catalyst. Org. Lett. 23 (7), 2742–2747. 10.1021/acs.orglett.1c00659 33754743 PMC8041384

